# *Arabidopsis* RALF4 Rapidly Halts Pollen Tube Growth by Increasing ROS and Decreasing Calcium Cytoplasmic Tip Levels

**DOI:** 10.3390/biom14111375

**Published:** 2024-10-29

**Authors:** Sofía C. Somoza, Noelia A. Boccardo, Franco Santin, Ana R. Sede, Diego L. Wengier, Aurélien Boisson-Dernier, Jorge P. Muschietti

**Affiliations:** 1 Instituto de Investigaciones en Ingeniería Genética y Biología Molecular, “Dr. Héctor Torres” (INGEBI-CONICET), Vuelta de Obligado 2490, Buenos Aires C1428ADN, Argentina; sofiacristina.somoza@unipd.it (S.C.S.); noeliaboccardo@gmail.com (N.A.B.); francosantin@gmail.com (F.S.); ana.sede@ibmp-cnrs.unistra.fr (A.R.S.); dwengier@stanford.edu (D.L.W.); 2 Departamento de Biodiversidad y Biología Experimental, Facultad de Ciencias Exactas y Naturales, Universidad de Buenos Aires, Intendente Güiraldes 2160, Ciudad Universitaria, Pabellón II, Buenos Aires C1428EGA, Argentina; 3 Institute for Plant Sciences, University of Cologne, 50674 Cologne, Germany

**Keywords:** peptide signaling, pollen tube, polar growth, synthetic peptides, *Arabidopsis*, calcium, ROS

## Abstract

In recent years, the rapid alkalinization factor (RALF) family of cysteine-rich peptides has been reported to be crucial for several plant signaling mechanisms, including cell growth, plant immunity and fertilization. RALF4 and RALF19 (RALF4/19) pollen peptides redundantly regulate the pollen tube integrity and growth through binding to their receptors ANXUR1/2 (ANX1/2) and Buddha’s Paper Seal 1 and 2 (BUPS1/2), members of the *Catharanthus roseus* RLK1-like (CrRLK1L) family, and, thus, are essential for plant fertilization. However, the signaling mechanisms at the cellular level that follow these binding events remain unclear. In this study, we show that the addition of synthetic peptide RALF4 rapidly halts pollen tube growth along with the excessive deposition of plasma membrane and cell wall material at the tip. The ratiometric imaging of genetically encoded ROS and Ca^2+^ sensors-expressing pollen tubes shows that RALF4 treatment modulates the cytoplasmic levels of reactive oxygen species (ROS) and calcium (Ca^2+^) in opposite ways at the tip. Thus, we propose that pollen RALF4/19 peptides bind ANX1/2 and BUPS1/2 to regulate ROS and calcium homeostasis to ensure proper cell wall integrity and control of pollen tube growth.

## 1. Introduction

The pollen tube is among the fastest growing cells in the plant kingdom; it grows in an apical, oscillatory and directional way, in order to reach the ovule and deliver the sperm cells for double fertilization. The main metabolic activity of the pollen tube consists in the synthesis of the materials needed for the continuous plasma membrane and cell wall assembly and deposition that occur at the tip, both necessary for this rapid and polar elongation. To this end, pollen tube growth is maintained by a complex and coordinated regulation of the actin cytoskeleton, endo- and exocytosis processes, ionic gradients of Ca^2+^, K^+^, Cl^−^ and H^+^ and reactive oxygen species (ROS) production [1].

Pollen tube growth is dependent on an oscillatory positive feedback loop of calcium ions (Ca^2+^), reactive oxygen species (ROS) and pH. The oscillations of ROS, Ca^2+^ and pH are coupled to a transient cell wall loosening at the tip, which is accompanied by the secretion of new cell wall materials. This enables a turgor-driven localized pollen tube tip expansion [1].

Rapid alkalinization factors (RALFs) are a family of small cysteine-rich secreted peptides that are present throughout the plant kingdom, with 34 members in *Arabidopsis thaliana*. The first RALF peptide was described as rapidly alkalinizing the media of tobacco suspension-cultured cells and inhibiting tomato and *Arabidopsis* primary root growth [2]; since then, they have been reported as key regulators in numerous and diverse physiological processes, working as signals that activate multiple receptors and co-receptors, and, subsequently, a myriad of cellular pathways [3,4,5].

RALF4 and RALF19 (RALF4/19) are crucial for the maintenance of pollen tube growth, through their interaction with the CrRLK1L family receptor-like kinases ANXUR1 and ANXUR2 (ANX1/2) and Buddha’s Paper Seal 1 and 2 (BUPS1/2) [6,7], forming a complex with two pollen LORELEI-Like-Glycosylphosphatidylinositol (GPI)-anchored proteins (LLG2/3) [7,8] and COBRA-like protein 11 (COBL11) [9]. These interactions activate a signaling pathway that includes a receptor-like cytoplasmic kinase (RLCK) called MARIS (MRI) [10] and pollen-expressed NADPH oxidases (respiratory burst oxidase homolog H and J, RBOH H/J), regulating the cytosolic levels of ROS and calcium [11,12]. On the other hand, RALF4 also interacts with leucine-rich repeat extensins (LRX8/9/10/11), chimeric proteins that participate in cell wall assembly during pollen tube growth [13]. Intriguingly, during growth, the pollen of knock-out and knock-down mutant plants corresponding to all the above-mentioned genes, including *ralf4-1* and *amiRRALF4/19* plants, burst prematurely to different extents. In contrast, the overexpression of ANX1/2 has the effect of inhibiting pollen tube growth [11]. This appears to be achieved by the overactivation of exocytosis followed by cell wall accumulation at the pollen tube tip. Recently, it has been shown that RALF4, in complex with LRX8, interacts with demethylesterified pectins, increasing cell wall strength in the pollen tube shank [14]. Moreover, the lack of RALF4/19 or LRX8/9/10/11 causes severe problems in maintaining the integrity of the pollen tube cell wall, triggering tip bursting [13,14,15,16,17]. Interestingly, it has been reported that the ovule-expressed RALF34 induces in vitro pollen tube bursting and binds in vitro to ANX1/2 and BUPS1/2, displacing RALF4/19 [18]. 

It is evident that the factors previously outlined play a role in regulating calcium dynamics and ROS levels in pollen tubes. However, the precise and immediate sequence of the cellular responses triggered by the application of RALF peptides and how it affects the dynamics of ROS and Ca^2+^ cytoplasmic levels at a single cell resolution remains unclear. In this work, we study how the different cellular processes involved in the regulation and maintenance of pollen tube growth are affected by the mature synthetic peptide RALF4. For this purpose, we monitor growing pollen tubes expressing the genetically encoded sensors of ROS (HyPer1) and calcium (YC3.60) upon treatment with RALFs and evaluate the content of de-methylated pectin in the cell wall [19].

## 2. Materials and Methods

### 2.1. Plant Material and Growth Conditions

Wild-type *Arabidopsis thaliana* plants used in this work correspond to the ecotype Columbia-0 (Col-0). The T-DNA insertion line *ralf4* of *Arabidopsis* (AT1G28270) and the *amiRRALF4/19* line were previously described [15] as the *anx1 anx2* double mutant line complemented with ANX1-YFP [11]. The RFP/GFP line was obtained by crossing the individual lines.

Seeds were surface sterilized in 70% ethanol for 30 min and in 96% ethanol for 5 min. For stratification, sterilized seeds were incubated in petri dishes with 0.5× MS (Murashige and Skoog) basal media at 4 °C for 2 days in dark and then transferred to a growth chamber at 22 °C under constant light and relative humidity maintained at 65%. Then, 10-day-old plants were transferred to soil and watered twice a week with Hakaphos 0.75 g/L water-soluble fertilizer (Compo Expert Argentina S.R.L, Buenos Aires, Argentina).

### 2.2. Stable Fusion Protein Expression in Arabidopsis Pollen Tubes

To generate the *pRALF4-RALF4-RFP*, *pRALF19-RALF19-GFP*, *pRALF4-rRALF4-RFP* and *pRALF19-rRALF19-GFP* constructs, Gateway cloning was used. The coding sequences for *RALF4*, *RALF19*, *rRALF4* and *rRALF19* were synthetized flanked by Gateway sites (NZYtech) and the promoter regions of *RALF4/19* were cloned into the *pENTR1a* vector [15]. These two types of vectors were recombined in pGWB659 (RFP) and pGWB650 (GFP) [20].

### 2.3. Synthetic Peptides

The RALF4, TAMRA-RALF4, RALF4 scrambled and RALF34 peptides (Table S1) were synthesized by Proteogenix (Schiltigheim, France) at the 15–19 mg scale with purity > 95% and dissolved in sterile pure water. All peptide treatments on growing pollen tubes were consistently performed at a concentration of 250 nM.

### 2.4. In Vitro Pollen Germination 

One-day open flowers were collected and incubated at 22 °C in a moisture incubation box for 30 min [13] and brushed onto slides containing 450 μL of fresh semi-solid pollen germination media (PGM) [21]. Slides in the moisture incubation box were pre-incubated for 30 min at 30 °C before returning them to 22 °C for 2–4 h. Pollen tubes were imaged using a confocal microscope (Leica TCS-SPE and SP8, Wetzlar, Germany). Pollen tubes that were at least twice the length of the pollen grain and displaying vesicle movement were considered to be healthy and actively growing.

For treating pollen tubes (with synthetic RALFs, FM4-64, propidium iodide), 100 μL of liquid PGM containing either 250 nM synthetic RALFs, 2 mM FM4-64 or 0.3 mM propidium iodide was applied on the semi-solid PGM for 5 min and washed away with fresh PGM before imaging. Dynamic changes were monitored right away. 

Fluorescence intensity (after background subtraction) and pollen tube length were measured using the ImageJ 1.53j software (Bethesda, MD, USA).

### 2.5. Measurement of Root Growth Inhibition by Synthetic RALFs

Sterilized wild-type seeds were incubated in square petri dishes with 0.5× MS media, stratified and then moved in vertical position to the growth chamber. After 4 days, seedlings were transferred to 12-well plates containing 4 mL of liquid 0.5× MS with or without 1 μM RALF4/RALF34/RALF4 scrambled for 4 days. Plants were placed on 0.5× MS media with extended roots and the main root length was measured using the ImageJ 1.53j software (Bethesda, MD, USA). 

### 2.6. Ratiometric Imaging 

Fluorescence in growing pollen tubes expressing either HyPer1 [22] or YC3.60 [23] sensors was acquired by confocal microscope (Leica SP8, Wetzlar, Germany) and quantified using ImageJ 1.53j software (Bethesda, MD, USA) as already described [11].

#### 2.6.1. HyPer1 Imaging

HyPer1 fluorescence was acquired with the sequential mode, exciting at 488 nm and collecting an emission between 500 and 540 nm for F_488_ and exciting at 405 nm and collecting at 500–540 nm for F_405_. F_488_/F_405_ ratiometric measurements were determined with ImageJ 1.53j software and its ROI Manager tool (Bethesda, MD, USA) after background subtraction.

#### 2.6.2. YC3.60 Imaging

YC3.60 was excited at 458 nm and emission was collected with the sequential mode at 469–501 nm and 522–554 nm for F_CFP_ and F_Venus_, respectively. F_CFP_/F_Venus_ was calculated, as described for HyPer1.

## 3. Results

### 3.1. RALF4 and RALF19 Peptides Colocalize in the Tip Region of the Pollen Tube

**Figure 1:**
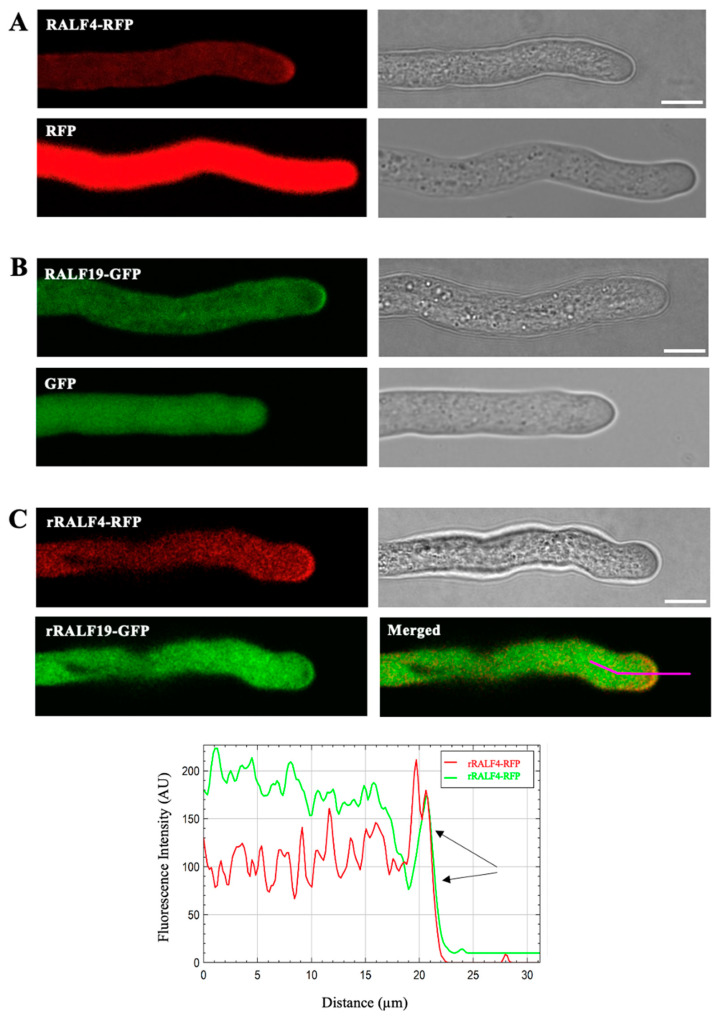
Subcellular localization of RALF4-RFP and RALF19-GFP in growing pollen tubes. A representative pollen tube from each homozygous line is shown. (**A**) Upper panels: *pRALF4*::*RALF4-RFP*; lower panels: *pRALF4*::*RFP* (control). (**B**) Upper panels: *pRALF19*::*RALF19-GFP*; lower panels: *pRALF19*::*GFP* (control), all of them in the Col-0 background. Scale bar: 10 µm. (**C**) RALF4 and RALF19 co-localize in the pollen tube tip (black arrows). A representative pollen tube from the *amiRRALF4/19* line expressing both *pRALF4::rRALF4-RFP* and *pRALF19::rRALF19-GFP* is shown. GFP and RFP fluorescence are shown with and without overlay (Merged). Scale bar: 10 µm. Fluorescence intensity of RFP and GFP were measured along a longitudinal line drawn from the cytoplasm to the tip.

We previously showed that *RALF4* and *RALF19* are specifically expressed in pollen and pollen tubes [15]. In order to study the subcellular localization of RALF4 and RALF19 in pollen tubes, we generated transgenic plants that express *pRALF4*::*RALF4-RFP* and *pRALF19*::*RALF19-GFP* in the wild-type Col-0 background and *pRALF4*::*RFP* and *pRALF19*::*GFP* were used as controls. As shown in Figure 1A,B, RALF4 and RALF19 fusion peptides localization signals appear less homogeneous than the fluorescent signal in the control lines, suggesting a granular localization in the cytoplasm and an enrichment at the periphery of the tip. This result suggests that RALF4/19 are transported in vesicles towards the apoplast of the pollen tube tip where they will be secreted. Note that the pollen of the *pRALF4*::*RALF4-RFP*- and *pRALF19*::*RALF19-GFP*-expressing plants showed lower germination, as has already been observed for *pLAT52*::*RALF4*-overexpressing plants [15], possibly caused by deregulated levels of RALF4 (Figure S1).

In order to analyze the co-localization of RALF4/19 in their corresponding mutant background, we expressed *RALF4-RFP* and *RALF19-GFP* in the *amiRRALF4/19* background [15]. Since *amiRRALF4/19* plants express the microRNA against *RALF4/19*, the *amiRRALF4/19*-resistant versions of RALF4 and RALF19 driven by their own promoters (*pRALF4*::*rRALF4-RFP* and *pRALF19*::*rRALF19-GFP*) were used [15]. Then, the line *amiRRALF4*/19 *pRALF4::rRALF4-RFP/pRALF19::rRALF19-GFP* was obtained by crossing the previously mentioned individual transgenic lines. RALF4 and RALF19 fusion peptides displayed the same localization patterns as in the Col-0 background and appeared to colocalize in the tip region of the pollen tube (Figure 1C).

### 3.2. Synthetic RALF4 Peptide Inhibits Pollen Tube Growth

To study the direct effect of RALF peptides on pollen tube dynamics, synthetic non-labeled RALF4, RALF4 labeled with the fluorophore 5-TAMRA (TAMRA-RALF4), RALF4 scrambled (same amino acids as RALF4 but in randomized position), and RALF34 peptides were used (Table S1). Within 50 s of adding TAMRA-RALF4, the TAMRA-derived fluorescence became first visible at the periphery of the pollen tube tip and, after 300 s, it was observed throughout the entire pollen tube and in fluorescent cytoplasmic granules, including the tube shanks where it has been shown to bind demethylesterified pectins and LRXs proteins [14]. This suggests that RALF4 peptides bind at the cell periphery and then become internalized (Figure S2A and Video S1). Interestingly, TAMRA-RALF4-treated tubes quickly stopped growing.

To confirm the inhibitory activity of the synthetic RALF4 on pollen tube growth, pollen tubes were treated with 250 nM RALF peptides and their growth was recorded for three minutes after the addition. RALF4 completely halted pollen tube growth (38 out of 40 pollen tubes stop growing immediately; Figure S2B), although the characteristic continuous vesicle movement and cytoplasmic streaming was still observed (Figure S2C and Video S2). In clear contrast, pollen tubes kept on growing after the addition of RALF34 (23 out of 23), RALF4 scrambled (30 out of 34) and pollen germination media (PGM, “Mock”) (37 out of 38), suggesting that the inhibitory action of RALF4 is specific and that the peptides RALF34 and RALF4 scrambled have no effect on pollen growth. In these experiments, the addition of the RALF34 peptide did not trigger any pollen tube bursting as reported previously [18].

To ensure that the lack of effect of the synthetic RALF34 peptide on pollen tube growth was not due to its inactivity, the root growth of *Arabidopsis* seedlings was studied, as it known to be inhibited by RALF34 but not by RALF4 [24,25]. Four-day-old wild-type Col-0 seedlings were grown in the presence of 1 μM RALF4, RALF34 and RALF4 scrambled, and the length of the main roots was measured after four days. The RALF34 treatment, but neither the RALF4 nor the RALF4 scrambled treatment, significantly inhibited root growth (Figure S3), thereby demonstrating that the synthetic RALF34 peptide is functional. Altogether, these results show that the synthetic peptides RALF4 and RALF34 are functional and have their own specific biological activity in pollen tubes and roots, respectively.

### 3.3. Synthetic RALF4 Triggers Cell Wall Accumulation and Plasma Membrane Retraction at the Tip of Pollen Tubes

**Figure 2:**
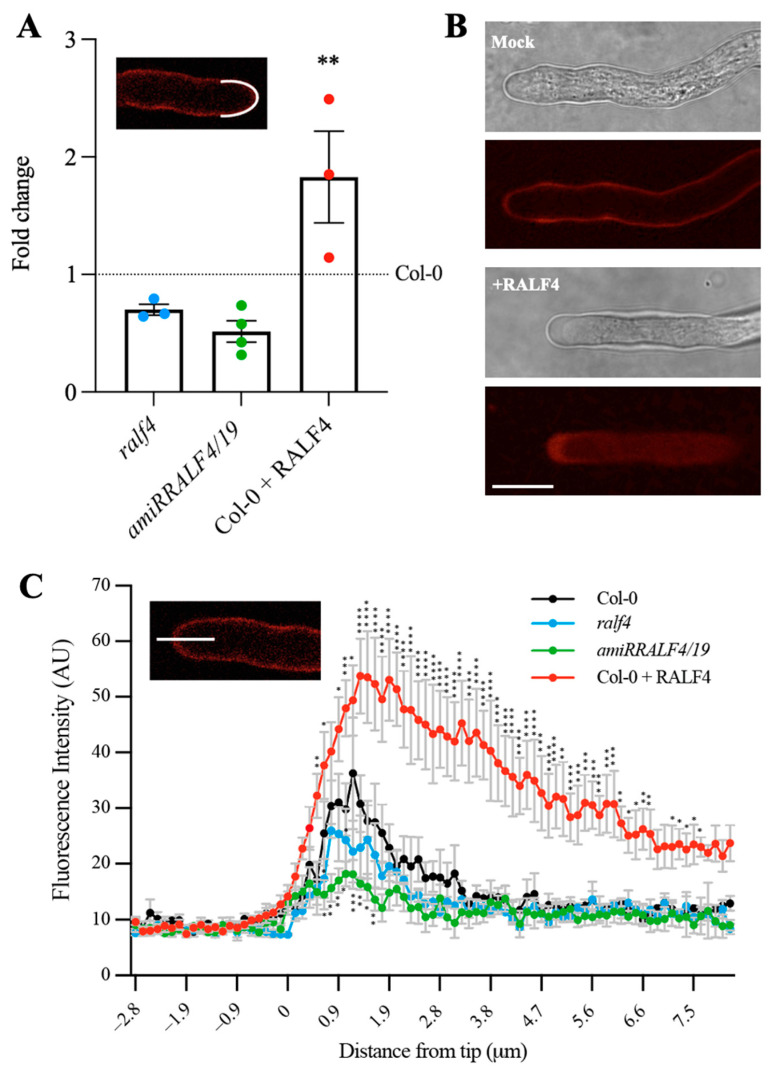
Cell wall pectin levels in RALF4-treated pollen tubes. Pollen tubes stained with propidium iodide (PI) from Col-0, *ralf4*, *amiRRALF4/19* lines and from Col-0 previously treated with synthetic RALF4 peptide (Col-0 + RALF4) are shown. (**A**) PI fluorescence intensity measured in the pollen tube tip, along the perimeter of the apical and subapical regions for *ralf4*, amiRALF4/19 and Col-0 pollen tubes relative to untreated Col-0 pollen tubes. Data are shown as the mean ± SEM of 3 independent experiments with n = 9–12 pollen tubes each. (**B**) Representative wild-type growing pollen tubes stained with PI, previously treated with RALF4 peptide or mock solution (PGM). Scale bar: 10 µm. (**C**) Fluorescence intensity of PI was measured along a longitudinal line from the tip to the cytoplasm. Data are shown as the mean ± SEM of five independent experiments with n = 9–12 pollen tubes each. (**A**,**C**) Asterisks indicate a significant difference (**A**) between Col-0 with and without RALF4 treatment and (**B**) between Col-0 and amiRRALF4/19, according to two-way ANOVA test followed by Tukey’s test: * *p* < 0.05, ** *p* < 0.01, *** *p* < 0.001 and **** *p* < 0.0001.

The regulation of the cell wall integrity in pollen tubes is essential for the control of their polar growth. In order to determine how RALF4 influences the cell wall, the levels of pectin, one of the main components of the pollen tube cell wall, were analyzed. For this, pollen tubes from *ralf4*, *amiRRALF4/19* and Col-0 plants, with or without previous incubation with the synthetic RALF4 peptide, were germinated in vitro and treated with propidium iodide (PI), which stains de-methylated pectins through binding to the carboxylic residues of non-carboxylated homogalacturonans [19]. The fluorescence intensity of PI was measured along the perimeter of the apical and subapical regions (Figure 2A) and along the pollen tube by tracing a longitudinal line from the tip into the cytoplasm (Figure 2C). Whereas *amiRRALF4/19* and, to a lesser extent, *ralf4* pollen tubes displayed a decrease in PI fluorescence intensity in the tip region, the RALF4 treatment led to a significant increase in PI fluorescence in wild-type pollen tubes (Figure 2B). This result suggests that, in wild-type pollen tubes, RALF4 participates positively in the deposition of pectin in the tip cell wall, necessary for the proper polar growth of pollen tubes. 

**Figure 3:**
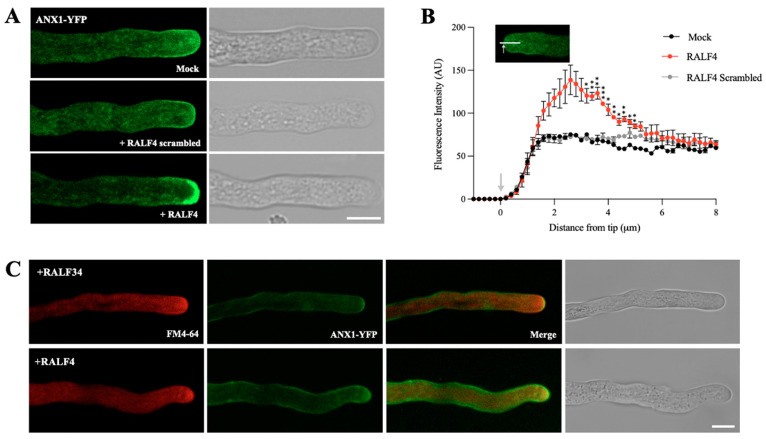
ANX1 localization in RALF4-treated pollen tubes. (**A**) Representative pollen tubes from *anx1 anx2* mutant expressing *ANX1-YFP* after treatments with PGM (Mock), 250 nM RALF4 or 250 nM RALF4 scrambled. Scale bar: 7 µm. (**B**) ANX1-YFP fluorescence intensity was measured along a longitudinal line from the tip to the cytoplasm. Data are shown as the mean ± SEM of three independent experiments with n = 7–9 pollen tubes each. Asterisks indicate a significant difference between RALF4 and mock treatments, according to two-way ANOVA test followed by Dunnet’s test: * *p* ≤ 0.05, ** *p* ≤ 0.01 and *** *p* ≤ 0.001. (**C**) Representative pollen tubes from *anx1 anx2* mutant expressing *ANX1-YFP* after treatments with 250 nM RALF34 and 250 nM RALF4 and stained with FM4-64. Scale bar: 10 µm.

It has been previously proposed that apoplastic RALF4 interacts with the extracellular domains of the receptor kinases ANX1/2 and BUPS1/2, activating a signaling cascade that controls pollen tube integrity and growth [5,15]. ANX1 overexpression was shown to inhibit pollen tube growth by triggering the overactive deposition of plasma membrane and cell wall material at the tip [11]. To study whether ANX1 localization is modified after the incubation with its ligand RALF4, pollen from an *anx1 anx2* double-mutant line complemented with *ANX1-YFP* [11] was germinated in vitro. ANX1-YFP was enriched at the plasma membrane of the pollen tube tip and also in particulate structures in the apical cytoplasm (Figure 3A and Figure S4), as previously described [11]. When the *anx1 anx2* pollen tubes expressing ANX1-YFP were treated with 250 nM RALF4, a region about 2 to 5 µm away from the tip showed higher fluorescence levels of ANX1-YFP than in the RALF4 scrambled or mock controls (Figure 3A,B and Figure S4). As already described [11], membrane invaginations were also observed in some pollen tubes treated with RALF4 (n = 14 out 23) but neither in tubes treated with the RALF4 scrambled peptide (n = 0 out 23) nor in mock-treated tubes (n = 0 out 26; Figure S4). In addition, staining with the lipophilic dye FM4-64 reveals that, in the RALF4-treated pollen tubes, ANX1-YFP remained in membranous elements in the tip, as it co-localized with FM4-64 (Figure 3C).

Altogether, these results indicate that incubation with synthetic RALF4 leads to an increased secretion of plasma membrane and cell wall material at the tip, which, in turn, leads to growth cessation. This effect is similar to the one observed with ANX1-YFP-overexpressing pollen tubes [11], although to a lesser extent, possibly because the activation by exogenous RALF4 treatment is transient and not permanent as it is in the pollen tubes overexpressing ANX1-YFP from the pollen germination onward. 

### 3.4. Synthetic RALF4 Increases Cytoplasmic ROS Levels and Decreases Cytoplasmic Calcium Levels in Pollen Tubes

**Figure 4:**
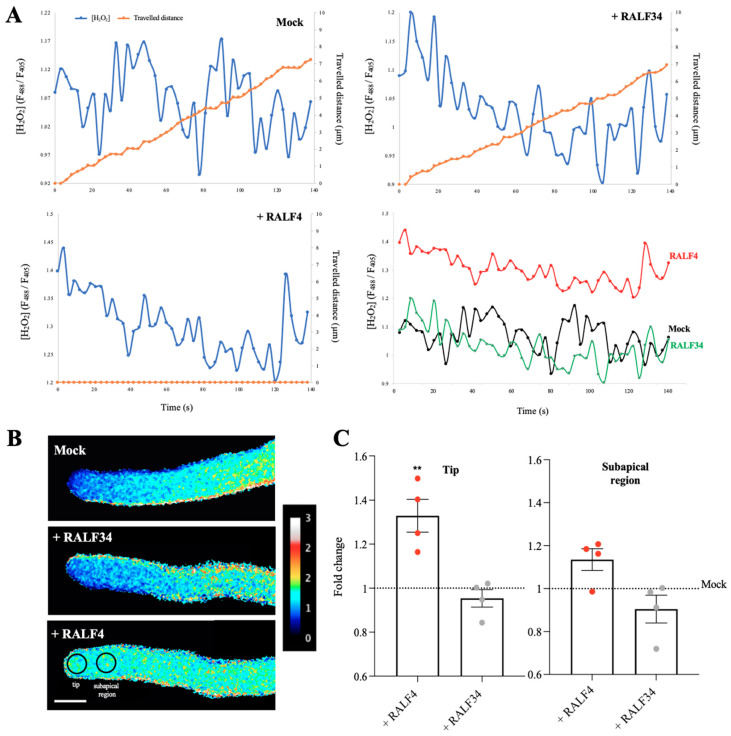
Cytoplasmic hydrogen peroxide levels in RALF4-treated pollen tubes. Col-0 pollen tubes expressing the cytosolic H_2_O_2_ sensor HyPer1 were used. (**A**) The distance traveled by the tip of a pollen tube (orange) and hydrogen peroxide levels in the cytoplasm of the tip region (blue) were measured over time after the addition of PGM (Mock) (**upper left**), RALF34 (**upper right**) or RALF4 (**lower left**). The **lower right** panel includes all H_2_O_2_ curves together to appreciate the differences. (**B**) Representative images of the tubes displaying the HyPer1 ratio (F_488_/F_405_) at the tip and subapical region. Scale bar: 10 µm. A representative pollen tube for each treatment is shown in (**A**,**B**). (**C**) Quantification of the HyPer1 ratio at the tip (**left panel**) and subapical region (**right panel**) of growing Col-0 pollen tubes after PGM (mock), RALF34 or RALF4 treatments. Data are shown as the mean ± SD of the fold change of the ratio obtained with RALF4 and RALF34 treatments with respect to mock, for four independent experiments. For each experiment, fluorescence was recorded in a 4 µm circular ROI every three seconds for three minutes, for seven to 11 pollen tubes per treatment. Double asterisks indicate a significant difference in comparison to mock treatment, according to one-way ANOVA test followed by a Tukey test (** *p* < 0.01).

**Figure 5:**
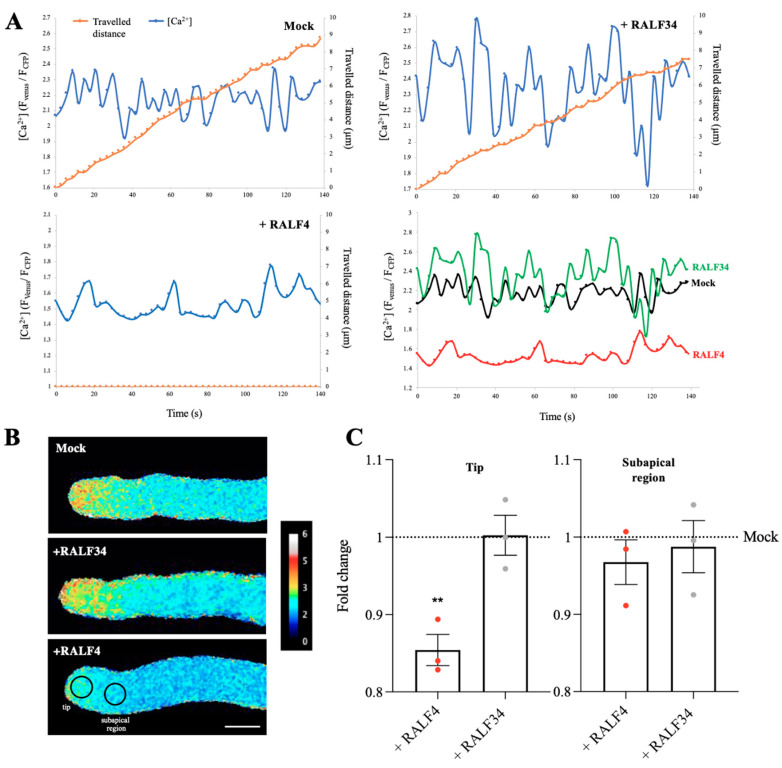
Cytoplasmic calcium levels in RALF4-treated pollen tubes. Col-0 pollen tubes expressing the cytosolic calcium sensor Yellow Cameleon 3.6 (YC3.60) were used. (**A**) The distance travelled by the tip of a pollen tube (orange) and the calcium levels in the cytoplasm of the tip region (blue) were measured over time after the addition of PGM (Mock) (**upper left**), RALF34 (**upper right**) and RALF4 (**lower left**). The **lower right** graph shows all H_2_O_2_ curves together to appreciate the differences. (**B**) Representative images of the tubes displaying the ratio F_CFP_/F_YFP_ at the tip and subapical region after the treatments. Scale bar: 10 µm. A representative pollen tube for each treatment is shown in (**A**,**B**). (**C**) Quantification of YC3.60 ratio (F_CFP_/F_YFP_) at the tip (**left panel**) and subapical region (**right panel**) of the pollen tubes after the treatments. Data are shown as the mean ± SD of the fold change of the ratio obtained with RALF4 and RALF34 treatments with respect to mock, for three independent experiments. For each experiment, fluorescence was recorded in a 4 µm circular ROI every three seconds for three minutes, for seven to eight pollen tubes per treatment. Double asterisks indicate a significant difference in comparison to mock treatment, according to one-way ANOVA test followed by a Tukey test (** *p* < 0.01).

Since the pollen-expressed ROS-producing NADPH oxidases RbohH and J have been genetically found to function downstream of the ANX1/2 receptors [11], we wondered what effect the addition of exogenous RALF4 on the concentration of cytoplasmic ROS in growing pollen tubes could have. To investigate this, plants expressing the genetically encoded biosensor HyPer1 [22], under the control of the pollen-specific LAT52 promoter, which detects hydrogen peroxide (H_2_O_2_), were used. No significant differences were found in cytoplasmic H_2_O_2_ levels in the selected ROI of the tube tips from wild-type and *ralf4-1* single-mutant backgrounds (Figure S5), suggesting that either RALF4 plays no role or that RALF4 and RALF19 are redundant in regulating ROS levels in pollen tubes. To discriminate between these two scenarios, we analyzed whether synthetic RALF4 directly affects cytoplasmic ROS dynamics in growing pollen tubes. Intriguingly, in RALF4-treated tubes, cytoplasmic H_2_O_2_ levels in the tip region were significantly higher than in wild-type tubes treated with RALF34 or with PGM (Figure 4A–C and Figure S6A and Videos S3A and S3B; all individual pollen tube recordings obtained in four independent experiments are displayed in Figure S7). Again, while wild-type pollen tubes treated with mock or with 250 nM RALF34 grew steadily without bursting, pollen tubes treated with 250 nM RALF4 stopped growing immediately (Figure 4A, Figure 5A and Figure S6A and Videos S3A and S3B). 

Moreover, because Ca^2+^ signaling has been shown to be regulated by RALF signaling [24], the effect of synthetic RALF4 on the apical concentration of cytoplasmic Ca^2+^ in pollen tubes expressing the ratiometric Yellow Cameleon 3.60 (YC3.60) biosensor [23] was analyzed. Remarkably, the addition of synthetic RALF4 at 250 nM caused a sharp decrease in the cytoplasmic calcium concentration in the apical region, but not in the subapical one, thereby almost abolishing the tip-focused calcium gradient (Figure 5A–C, Figure S6B, S8 and Videos S4A and S4B). As observed before, all the RALF4-treated pollen tubes stopped growing. In clear contrast, the mock and RALF34 treatment did not impact cytoplasmic calcium levels in YC3.60-expressing pollen tubes, the latter displaying expected tip-focus calcium gradient and steady growth (Figure 5A–C, Figure S6B and Videos S4A and S4B; all individual pollen tube recordings obtained in three independent experiments are displayed in Figure S8). Our results show that treatment with the synthetic RALF4, but not RALF34, rapidly perturbs ROS and Ca^2+^ homeostasis at the tip of pollen tubes.

All together, these results suggest that exogenously applied RALF4 synthetic peptide increases cytoplasmic tip H_2_O_2_ levels, decreases cytoplasmic tip calcium concentration and triggers cell wall accumulation and plasma membrane retraction, thereby rapidly halting pollen tube growth. This is consistent with the signaling function of RALF4 during pollen tube growth in which RALF4 molecules that do not interact with LRX and pectins are free to bind ANXs/BUPS, initiating a signaling cascade that would regulate ROS and Ca^2+^ levels, leading to the control of actin dynamics and cell wall deposition during pollen tube growth.

## 4. Discussion

Pollen tube growth is highly regulated through the establishment of an apical calcium gradient, actin polymerization and the generation of ROS. This growth also requires a malleable cell wall in the tip region to allow expansion without bursting and a constantly rigid enough cell wall at the shank to withstand lateral turgor pressures.

Here, we show that RALF4-RFP and RALF19-GFP peptides colocalize in the pollen tube tip region. This result agrees with the report of Zhou and colleagues [26] that shows that RALF4-GFP is secreted to the apoplast of the pollen tube tip, and with Mecchia et al., 2017 [15], which shows that synthetic peptides FITC-RALF4 and FITC-RALF19 bind to pollen tubes, with a more intense signal at the tip periphery. 

On one hand, we show that synthetic peptide RALF4 halts pollen tube growth as previously described [15,24]. Although growth cessation is extremely rapid, vesicles movement and cytoplasmic streaming in the tube continues (see Figure S2). On the other hand, as shown by Mecchia et al., 2017 [15], *ralf4* mutant pollen tubes in vitro initially grow faster than wild-type tubes and then burst. In essence, RALF4 would control the speed limit of the pollen tubes in order to maintain a proper growth. This growth inhibitory effect was specific to RALF4, as the addition of neither RALF4 scrambled nor synthetic RALF34, an ovule-expressed RALF peptide and ligand of THESEUS1 that regulates lateral root initiation [27], although previously reported to trigger pollen tube bursting [18], had any effect on pollen tube growth in our experimental conditions. This synthetic RALF34 peptide was functional, though, as it did inhibit root growth (see Figure S3), as previously reported [24,25].

Moreover, we found that synthetic RALF4 treatment increases pectin accumulation in the tip apoplast, augmenting cell wall thickness, which is likely the cause of pollen tube growth arrest. This correlates well with the fact that ANX1 overexpression also induces the overaccumulation of pectin at the tip and inhibits pollen tube growth [11]. When the cell wall of the tip reaches a thickness that does not allow further expansion, cytoplasmic movement and ongoing exocytosis lead to membrane retraction and, in some cases, to membrane invagination. This was described for pollen tubes overexpressing *ANX1-YFP* [11] and, in some cases, in this work, with synthetic RALF4 but not with the RALF4 scrambled peptide (see Figure S4). In addition, our findings are compatible with a recent report that states that RALF1 treatment induces cell wall swelling and plasma membrane invagination in root epidermal cells and binds to de-methylesterified pectin on the root surface, a process that is crucial for the RALF1-FERONIA association, which regulates extracellular matrix and plasma membrane dynamics [28]. In light of our findings, we postulate that a comparable regulatory mechanism may also occur at the pollen tube tip.

We also found that, after incubation with synthetic RALF4, the ANX1-YFP signal decreases at the margins of the tip and increases 3 μm away from the tip. The staining with FM4-64 confirms that the ANX1-YFP signal remains within the FM4-64-stained domain at the pollen tube tip that includes both the plasma membrane and the secretory vesicles. These observations seem to support the reported RALF1/23-triggered endocytosis of various cell surface regulators and the uptake increase of FM4-64 into the cytoplasm of root cells [29].

Our results show that the in vitro addition of synthetic RALF4 increases cytoplasmic H_2_O_2_ levels in the tip of the pollen tube, which immediately arrests its growth. This is consistent with a previous report that the overexpression of ANX1 is responsible for increased ROS production leading to pollen tube arrest [11]. Lassig and collaborators (2014) [12] proposed that NAD(P)H oxidases control tube growth speed; this aligns well with our results, which suggest that RALF4 is responsible, through the activation of ANX1/2 and NAD(P)H oxidases, for maintaining a balance between rapid and unstable growth leading to pollen tube bursting (*ralf4* mutant pollen tubes) and slow growth leading to pollen tube arrest (synthetic peptide RALF4 addition).

Gao and colleagues [30] reported a calcium increase in GCaMP6s-expressing pollen tubes incubated with insect-cell purified RALF4 peptide at 500 nM, but, unfortunately, did not monitor growth and calcium levels overtime. This result is opposite to what is shown here with our synthetic RALF4 peptide at 250 nM treatment that led to the decrease in Ca^2+^ levels specifically at the tip, almost abolishing the tip-focused Ca^2+^ gradient. Perhaps the different external calcium concentrations in the pollen germination media (2 mM Ca^2+^ in Gao et al., 2023 [30], while 5 mM in our study), or the fact that peptides obtained by expression in insect cells would be glycosylated, whereas pure synthetic peptides are not, could explain this difference. Nonetheless, there are several reports where synthetic RALF peptides have been shown to be functional in various cellular contexts [15,25]. Moreover, pollen tubes not only stop growing when there is a high calcium concentration along the tube, but also when calcium concentration decreases in the tube and, thus, the tip-focused gradient disappears [31]. Interestingly, another study has shown that treatment with recombinant RALF4 peptide inhibited pollen germination but failed to induce a cytosolic Ca^2+^ increase in 4-day-old seedlings, as opposed to treatment with RALF1 [24]. It is important to note that all RALF treatments in our study were carried out at the same peptide concentration (250 nM) on healthy, growing pollen tubes, as demonstrated in Video S2. Moreover, calcium and hydrogen peroxide levels were monitored over time using genetically encoded ratiometric sensors, eliminating the need for snapshots of immunolabeled or stained tubes, methods that can generate irreversible activities and artifacts.

It is important to consider the potential influence of alterations in cytoplasmic pH on HyPer1 recordings, given that Hyper1 activity has been observed to artificially increase in response to a rise in pH [32,33], and that pollen tubes exhibit an alkaline band in the subapical region and an acidic tip [34]. Therefore, we employed healthy and actively growing pollen tubes and documented the distance traversed by the pollen tubes during the experiments (See Figure 4 and Figure 5 and Supplementary Videos) and only measured HyPer1 activity in the tip and subapical region of pollen tubes known to be acidic [34]. This shows, as expected, the normal growth of mock- and RALF34-treated tubes but not of RALF4-treated tubes. The Cameleon calcium sensor YC3.60 is capable of accurately measuring even slight changes in the cpVenus/ECFP ratio with a high degree of certainty [32,35]. This enables the precise identification of the FRET and calcium values. Despite the pH sensitivity of ECFP and cpVenus, the FRET ratio remains stable when the physiological cytosolic pH is within a limited range, as is the case in the pollen tube tip.

Our study provides new evidence about how RALF4/19 would regulate pollen tube growth. Although RALF4/19 have been reported to be necessary to maintain proper pollen tube growth, at least in vitro [15,18], they are also involved in pollen tube reception by the synergids [30]. Synthetic or *E. coli*-expressed RALF4 bind to the FER–LRE–NTA protein complex of the filiform apparatus and induce Ca^2+^ spiking in the synergid cytoplasm, similarly to how arriving pollen tubes act [36]. Other pollen-expressed RALFs (RALF6, RALF7, RALF16, RALF36 and RALF37) have also been reported to interact in vitro with FER, ANJEA (ANJ) and HERCULES RECEPTOR KINASE 1 (HERK1) to act in polytube blocking and pollen tube reception [37]. One of them, RALF37, similar to RALF4 and RALF19, also triggered synergid Ca^2+^ spiking and activated the FER–LRE–NTA protein complex [36].

All these results suggest that RALF4/19 take part in controlling most of the aspects that regulate pollen–pistil interactions and fertilization. The function of the other RALFs expressed in pollen (RALF8, RALF9, RALF15, RALF25, RALF26 and RALF30) [38] remains to be determined, but what is clear is that none of them can substitute for the absence of RALF4/19 during pollination and fertilization.

Gao and colleagues [30] showed that RALF4, RALF19 and RALF34 bind FER in vitro. Just as RALF34 in vitro displaces RALF4 from binding to ANX1 [18], it is necessary to investigate whether there is also competition between pollen and ovule-derived RALFs in their binding to FER. It is tempting to think that pollen tubes-derived RALF4/19 could compete with and displace RALF34 from synergid-localized FER to initiate the calcium spiking in the synergids. Even though, in our hands, RALF34 did not trigger pollen tube bursting in vitro, during the in vivo intimate male–female gametophytic dialogue, RALF34 could bind to pollen-localized ANX/BUPS to induce the pollen tube tip bursting inside the receiving synergid cell [18]. This is a step that then should occur after RALF4/19 binding to FER and synergid Ca^2+^ spiking. Considering that RALF34 binds to FER at least in vitro, but cannot increase [Ca^2+^]_cyt_ and FER-LRE-NTA activity, it remains unclear what the function of the RALF34-FER interaction is in synergids. Unfortunately, the study of the *ralf34-1* loss-of-function mutant that did not display obvious fertilization-related phenotypes did not provide us with further clues [18]. It remains to be understood how the calcium spiking is orchestrated and how RALFs, CrRLK1Ls and LLGs, as well as the calcium channels of the MLO [30,39], CNGC [39] and OSCA [40] families, are precisely involved. 

This work, together with previous reports [6,8,11,15,18], confirms that RALF4 triggers a signaling cascade through LLG/ANX/BUPS receptor complexes that regulate cytosolic levels of ROS and calcium to control cell wall integrity during pollen tube growth. Unexpectedly, it has recently been reported that RALF4 has also a structural function during pollen tube growth [14]. The LRX8-RALF4 complex interacts specifically through RALF4 with demethylesterified pectins conferring strength to the cell wall at the shank of the tube, necessary for lateral cell wall stability during pollen tube growth. Therefore, only free RALF4 would bind to the LLG/ANX/BUPS receptor complexes at the tip, regulating pollen tube expansion. In addition to this, Liu and colleagues [29] demonstrated that RALF1 binds to pectin and, together, they recruit FER into a pectin–protein complex, thus initiating RALF-triggered signaling in the cell. It is tempting to consider that the strength of this signal at the tip is conditioned by the free concentration of RALF4 in the apoplast, which would serve as a proxy for the hazardousness of the cell wall integrity.

## 5. Conclusions

In plants, the RALF peptides play a significant role in regulating various physiological processes, interacting with multiple membrane receptors and triggering diverse cellular pathways. In this context, *Arabidopsis* RALF4 has been identified as a key factor in various aspects of plant fertilization but, often, was studied with treatments at different concentrations in different contexts. Here, using one constant concentration, our findings indicate that RALF4 is responsible for maintaining an optimal pollen tube growth rate by modulating, in opposing ways, the cytoplasmic levels of reactive oxygen species (ROS) and calcium (Ca^2+^) at the apical zone of pollen tubes.

## Data Availability

The data are in the manuscript or in the supplemental data.

## Supplementary Materials

The following supporting information can be downloaded at: https://www.mdpi.com/article/10.3390/biom14111375/s1, Figure S1: In vitro pollen germination of two independent lines (#1 and #2) of wild-type plants expressing the RALF4-RFP fusion driven by the RALF4 promoter; Figure S2: Synthetic RALF4 peptide activity on pollen tube growth; Figure S3: Root growth inhibition by synthetic RALF34 peptide; Figure S4: Localization of ANX1-YFP in pollen tubes. Figure S5: Hydrogen peroxide levels in Col-0 and *ralf4* mutant pollen tubes; Figure S6: H_2_O_2_ and Ca^2+^ levels over time along the tube after RALF treatments; Figure S7: Raw data for Figure 4, which details the cytoplasmic hydrogen peroxide levels in all mock and RALF4/34-treated pollen tubes monitored over four independent experiments; Figure S8: Raw data for Figure 5, which details the cytoplasmic calcium levels in all mock and RALF4/34-treated pollen tubes monitored over three independent experiments; Table S1: Amino acid sequence of the synthetic peptides used in this work; Video S1: Wild-type growing pollen tube after the addition of TAMRA-RALF4; Video S2: Wild-type growing pollen tubes before and after the addition of RALF4 peptide; Video S3: Wild-type growing pollen tube expressing cytosolic HyPer1 after the addition of PGM (Mock) and RALF4; Video S4: Wild-type growing pollen tube expressing YC3.60 after the addition of PGM (Mock) and RALF4.

## References

[1] Johnson M.A., Harper J.F., Palanivelu R. (2019). A Fruitful Journey: Pollen Tube Navigation from Germination to Fertilization. Annu. Rev. Plant Biol..

[2] Pearce G., Moura D.S., Stratmann J., Ryan C.A. (2001). RALF, a 5-kDa Ubiquitous Polypeptide in Plants, Arrests Root Growth and Development. Proc. Natl. Acad. Sci. USA.

[3] Murphy E., Smet I.D. (2014). Understanding the RALF Family: A Tale of Many Species. Trends Plant Sci..

[4] Blackburn M.R., Haruta M., Moura D.S. (2020). Twenty Years of Progress in Physiological and Biochemical Investigation of RALF Peptides. Plant Physiol..

[5] Somoza S.C., Sede A.R., Boccardo N.A., Muschietti J.P. (2021). Keeping up with the RALFs: How These Small Peptides Control Pollen–Pistil Interactions in *Arabidopsis*. New Phytol..

[6] Ge Z., Zhao Y., Liu M.-C., Zhou L.-Z., Wang L., Zhong S., Hou S., Jiang J., Liu T., Huang Q. (2019). LLG2/3 Are Co-Receptors in BUPS/ANX-RALF Signaling to Regulate *Arabidopsis* Pollen Tube Integrity. Curr. Biol..

[7] Zhu L., Chu L.-C., Liang Y., Zhang X.-Q., Chen L.-Q., Ye D. (2018). The *Arabidopsis* CrRLK1L Protein Kinases BUPS1 and BUPS2 Are Required for Normal Growth of Pollen Tubes in the Pistil. Plant J. Cell Mol. Biol..

[8] Feng H., Liu C., Fu R., Zhang M., Li H., Shen L., Wei Q., Sun X., Xu L., Ni B. (2019). LORELEI-LIKE GPI-ANCHORED PROTEINS 2/3 Regulate Pollen Tube Growth as Chaperones and Coreceptors for ANXUR/BUPS Receptor Kinases in *Arabidopsis*. Mol. Plant.

[9] Li H., Yang Y., Zhang H., Li C., Du P., Bi M., Chen T., Qian D., Niu Y., Ren H. (2023). The *Arabidopsis* GPI-Anchored Protein COBL11 Is Necessary for Regulating Pollen Tube Integrity. Cell Rep..

[10] Boisson-Dernier A., Franck C.M., Lituiev D.S., Grossniklaus U. (2015). Receptor-like Cytoplasmic Kinase MARIS Functions Downstream of CrRLK1L-Dependent Signaling during Tip Growth. Proc. Natl. Acad. Sci. USA.

[11] Boisson-Dernier A., Lituiev D.S., Nestorova A., Franck C.M., Thirugnanarajah S., Grossniklaus U. (2013). ANXUR Receptor-like Kinases Coordinate Cell Wall Integrity with Growth at the Pollen Tube Tip via NADPH Oxidases. PLoS Biol..

[12] Lassig R., Gutermuth T., Bey T.D., Konrad K.R., Romeis T. (2014). Pollen Tube NAD(P)H Oxidases Act as a Speed Control to Dampen Growth Rate Oscillations during Polarized Cell Growth. Plant J. Cell Mol. Biol..

[13] Sede A.R., Borassi C., Wengier D.L., Mecchia M.A., Estevez J.M., Muschietti J.P. (2018). *Arabidopsis* Pollen Extensins LRX Are Required for Cell Wall Integrity during Pollen Tube Growth. FEBS Lett..

[14] Moussu S., Lee H.K., Haas K.T., Broyart C., Rathgeb U., De Bellis D., Levasseur T., Schoenaers S., Fernandez G.S., Grossniklaus U. (2023). Plant Cell Wall Patterning and Expansion Mediated by Protein-Peptide-Polysaccharide Interaction. Science.

[15] Mecchia M.A., Santos-Fernandez G., Duss N.N., Somoza S.C., Boisson-Dernier A., Gagliardini V., Martínez-Bernardini A., Fabrice T.N., Ringli C., Muschietti J.P. (2017). RALF4/19 Peptides Interact with LRX Proteins to Control Pollen Tube Growth in *Arabidopsis*. Science.

[16] Fabrice T.N., Vogler H., Draeger C., Munglani G., Gupta S., Herger A.G., Knox P., Grossniklaus U., Ringli C. (2018). LRX Proteins Play a Crucial Role in Pollen Grain and Pollen Tube Cell Wall Development. Plant Physiol..

[17] Wang X., Wang K., Yin G., Liu X., Liu M., Cao N., Duan Y., Gao H., Wang W., Ge W. (2018). Pollen-Expressed Leucine-Rich Repeat Extensins Are Essential for Pollen Germination and Growth. Plant Physiol..

[18] Ge Z., Bergonci T., Zhao Y., Zou Y., Du S., Liu M.-C., Luo X., Ruan H., García-Valencia L.E., Zhong S. (2017). *Arabidopsis* Pollen Tube Integrity and Sperm Release Are Regulated by RALF-Mediated Signaling. Science.

[19] Rounds C.M., Lubeck E., Hepler P.K., Winship L.J. (2011). Propidium Iodide Competes with Ca^2+^ to Label Pectin in Pollen Tubes and *Arabidopsis* Root Hairs. Plant Physiol..

[20] Nakagawa T., Kurose T., Hino T., Tanaka K., Kawamukai M., Niwa Y., Toyooka K., Matsuoka K., Jinbo T., Kimura T. (2007). Development of Series of Gateway Binary Vectors, pGWBs, for Realizing Efficient Construction of Fusion Genes for Plant Transformation. J. Biosci. Bioeng..

[21] Boavida L.C., McCormick S. (2007). Temperature as a Determinant Factor for Increased and Reproducible in Vitro Pollen Germination in *Arabidopsis* Thaliana. Plant J. Cell Mol. Biol..

[22] Hernández-Barrera A., Velarde-Buendía A., Zepeda I., Sanchez F., Quinto C., Sánchez-Lopez R., Cheung A.Y., Wu H.-M., Cardenas L. (2015). Hyper, a Hydrogen Peroxide Sensor, Indicates the Sensitivity of the *Arabidopsis* Root Elongation Zone to Aluminum Treatment. Sensors.

[23] Nagai T., Yamada S., Tominaga T., Ichikawa M., Miyawaki A. (2004). Expanded Dynamic Range of Fluorescent Indicators for Ca(2+) by Circularly Permuted Yellow Fluorescent Proteins. Proc. Natl. Acad. Sci. USA.

[24] Morato do Canto A., Ceciliato P.H.O., Ribeiro B., Ortiz Morea F.A., Franco Garcia A.A., Silva-Filho M.C., Moura D.S. (2014). Biological Activity of Nine Recombinant AtRALF Peptides: Implications for Their Perception and Function in *Arabidopsis*. Plant Physiol. Biochem..

[25] Abarca A., Franck C.M., Zipfel C. (2021). Family-Wide Evaluation of RAPID ALKALINIZATION FACTOR Peptides. Plant Physiol..

[26] Zhou X., Lu J., Zhang Y., Guo J., Lin W., Van Norman J.M., Qin Y., Zhu X., Yang Z. (2021). Membrane Receptor-Mediated Mechano-Transduction Maintains Cell Integrity during Pollen Tube Growth within the Pistil. Dev. Cell.

[27] Gonneau M., Desprez T., Martin M., Doblas V.G., Bacete L., Miart F., Sormani R., Hématy K., Renou J., Landrein B. (2018). Receptor Kinase THESEUS1 Is a Rapid Alkalinization Factor 34 Receptor in *Arabidopsis*. Curr. Biol..

[28] Rößling A.-K., Dünser K., Liu C., Lauw S., Rodriguez-Franco M., Kalmbach L., Barbez E., Kleine-Vehn J. (2024). Pectin Methylesterase Activity Is Required for RALF1 Peptide Signalling Output. eLife.

[29] Liu M.-C.J., Yeh F.-L.J., Yvon R., Simpson K., Jordan S., Chambers J., Wu H.-M., Cheung A.Y. (2024). Extracellular Pectin-RALF Phase Separation Mediates FERONIA Global Signaling Function. Cell.

[30] Gao Q., Wang C., Xi Y., Shao Q., Hou C., Li L., Luan S. (2023). RALF Signaling Pathway Activates MLO Calcium Channels to Maintain Pollen Tube Integrity. Cell Res..

[31] Barberini M.L., Sigaut L., Huang W., Mangano S., Juarez S.P.D., Marzol E., Estevez J., Obertello M., Pietrasanta L., Tang W. (2018). Calcium Dynamics in Tomato Pollen Tubes Using the Yellow Cameleon 3.6 Sensor. Plant Reprod..

[32] Choi W.-G., Swanson S.J., Gilroy S. (2012). High-Resolution Imaging of Ca^2+^, Redox Status, ROS and pH Using GFP Biosensors. Plant J..

[33] Martin R.E., Postiglione A.E., Muday G.K. (2022). Reactive Oxygen Species Function as Signaling Molecules in Controlling Plant Development and Hormonal Responses. Curr. Opin. Plant Biol..

[34] Michard E., Dias P., Feijó J. (2008). Tobacco Pollen Tubes as Cellular Models for Ion Dynamics: Improved Spatial and Temporal Resolution of Extracellular Flux and Free Cytosolic Concentration of Calcium and Protons Using pHluorin and YC3.1 CaMeleon. Sex. Plant Reprod..

[35] Grenzi M., Resentini F., Vanneste S., Zottini M., Bassi A., Costa A. (2021). Illuminating the Hidden World of Calcium Ions in Plants with a Universe of Indicators. Plant Physiol..

[36] Gao Q., Wang C., Xi Y., Shao Q., Li L., Luan S. (2022). A Receptor-Channel Trio Conducts Ca^2+^ Signalling for Pollen Tube Reception. Nature.

[37] Zhong S., Li L., Wang Z., Zengxiang G., Li Q., Bleckmann A., Wang J., Song Z., Shi Y., Liu T. (2022). RALF Peptide Signaling Controls the Polytubey Block in *Arabidopsis*. Science.

[38] Loraine A.E., McCormick S., Estrada A., Patel K., Qin P. (2013). RNA-Seq of *Arabidopsis* Pollen Uncovers Novel Transcription and Alternative Splicing. Plant Physiol..

[39] Meng J.-G., Liang L., Jia P.-F., Wang Y.-C., Li H.-J., Yang W.-C. (2020). Integration of Ovular Signals and Exocytosis of a Ca^2+^ Channel by MLOs in Pollen Tube Guidance. Nat. Plants.

[40] Pei S., Tao Q., Li W., Qi G., Wang B., Wang Y., Dai S., Shen Q., Wang X., Wu X. (2024). Osmosensor-Mediated Control of Ca^2+^ Spiking in Pollen Germination. Nature.

