# Supplementary material for: Arabidopsis RALF4 Rapidly Halts Pollen Tube Growth by Increasing ROS and Decreasing Calcium Cytoplasmic Tip Levels
Source: Biomolecules. 2024 Oct 29;14(11):1375. doi: 10.3390/biom14111375 (PMC11591785; doi:10.3390/biom14111375)
Supplement: Supplementary file 1 [file biomolecules-14-01375-s001.zip › RS_SUPPLEMENTARY INFORMATION_biomolecules.pdf]

SUPPLEMENTARY INFORMATION

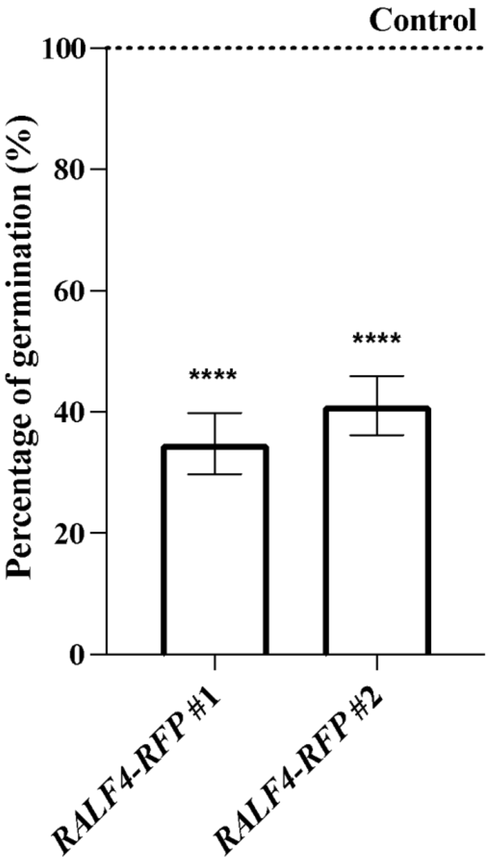

Supplementary Figure S1: *In vitro* pollen germination of two independent lines (#1 and #2) of wild-type plants expressing the RALF4-RFP fusion driven by the RALF4 promoter. The results are expressed as the percentage of germinated pollen grains respect to control plants (expressing RFP under the RALF4 promoter) from three independent experiments; for each experiment, between 200 and 600 pollen grains from 5 plants per genotype were counted. Pollen grains were classified as germinated when they showed a pollen tube at least twice the length of the pollen grain. Asterisks indicate a significant difference between *pRALF4::RALF4-RFP* #1 or #2 and control wild-type plants, according to two-way ANOVA **** $p < 0.0001$.

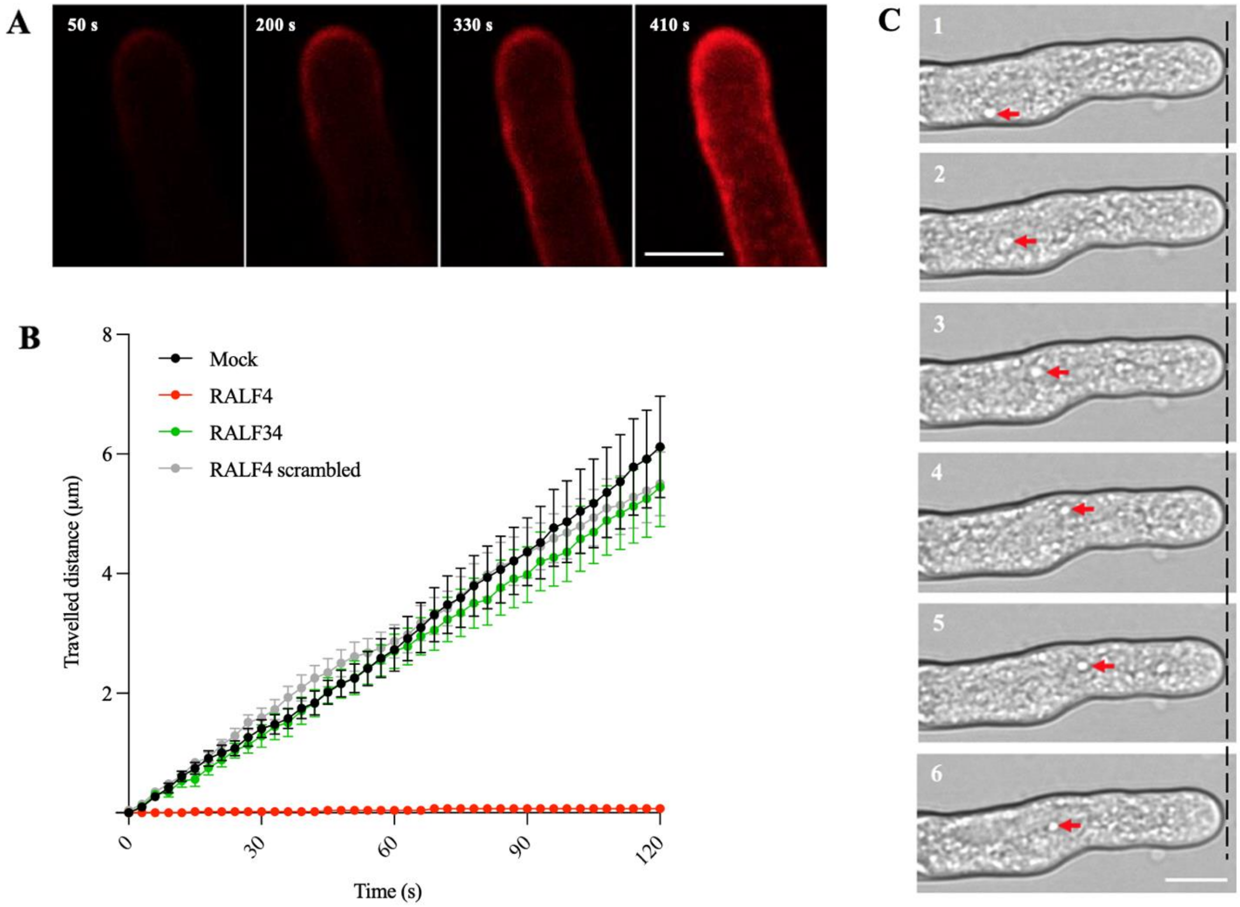

Supplementary Figure S2: Synthetic RALF4 peptide activity on pollen tube growth. (**A**) A representative wild-type pollen tube grown *in vitro* and treated with TAMRA-RALF4 is shown at 50 seconds, 200 seconds, 330 seconds and 410 seconds after treatment. Scale bar: 7 µm (**B**) Traveled distance over time of pollen tubes grown *in vitro* and treated with 250 nM synthetic peptides RALF4, RALF4 scrambled, RALF34 and liquid pollen germination media (PGM) as mock. The position of the pollen tube tip was registered every 3 seconds for 3 minutes. Data is shown as the mean ± SEM of 8 wild-type tubes per treatment. (**C**) Cytoplasmic trafficking in an arrested pollen tube after treatment with synthetic RALF4. The red arrow indicates the position of the same vesicle over 2 minutes. Scale bar: 10 µm.

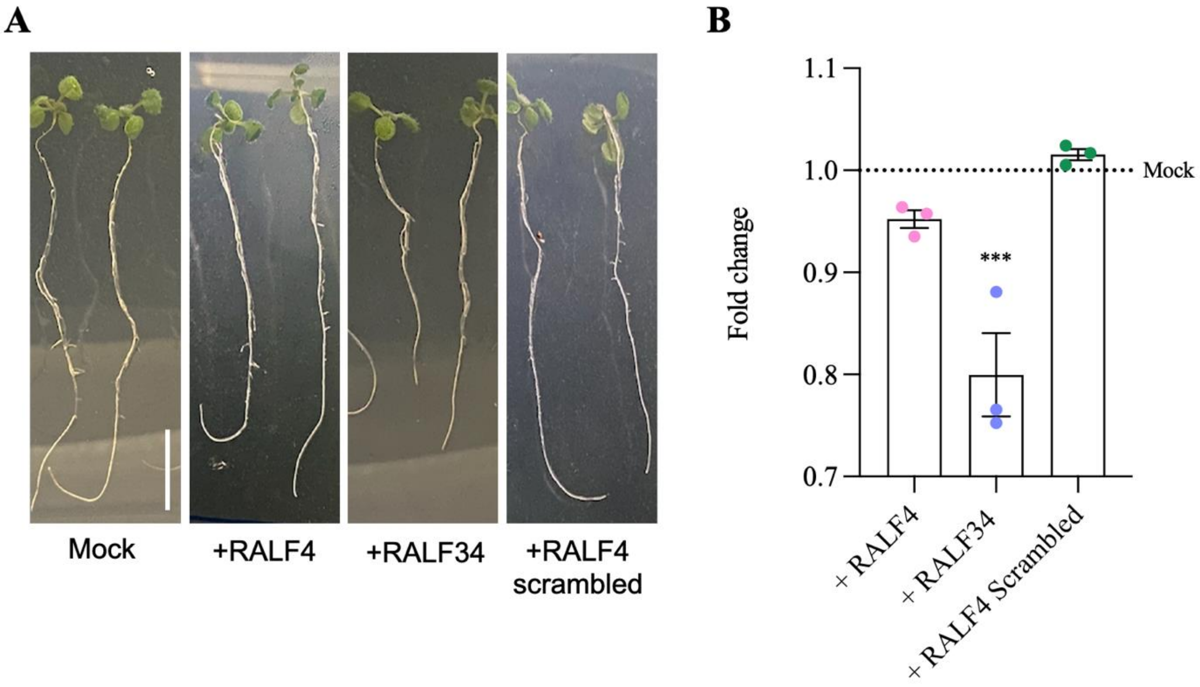

Supplementary Figure S3: Root growth inhibition by synthetic RALF34 peptide. (**A**) Wild-type seedlings treated with a mock solution (0,5X MS) and 1 μM RALF4, RALF34 or RALF4 scrambled synthetic peptides. Scale bar: 5mm. (**B**) Primary root length (cm). Data is shown as the mean ± SEM of three independent experiments with n= 10 seedlings each. Asterisks indicate a significant difference between mock and RALF34 treatment, according to one-way ANOVA test followed by Dunnett's test: ** $p < 0{,}01$.

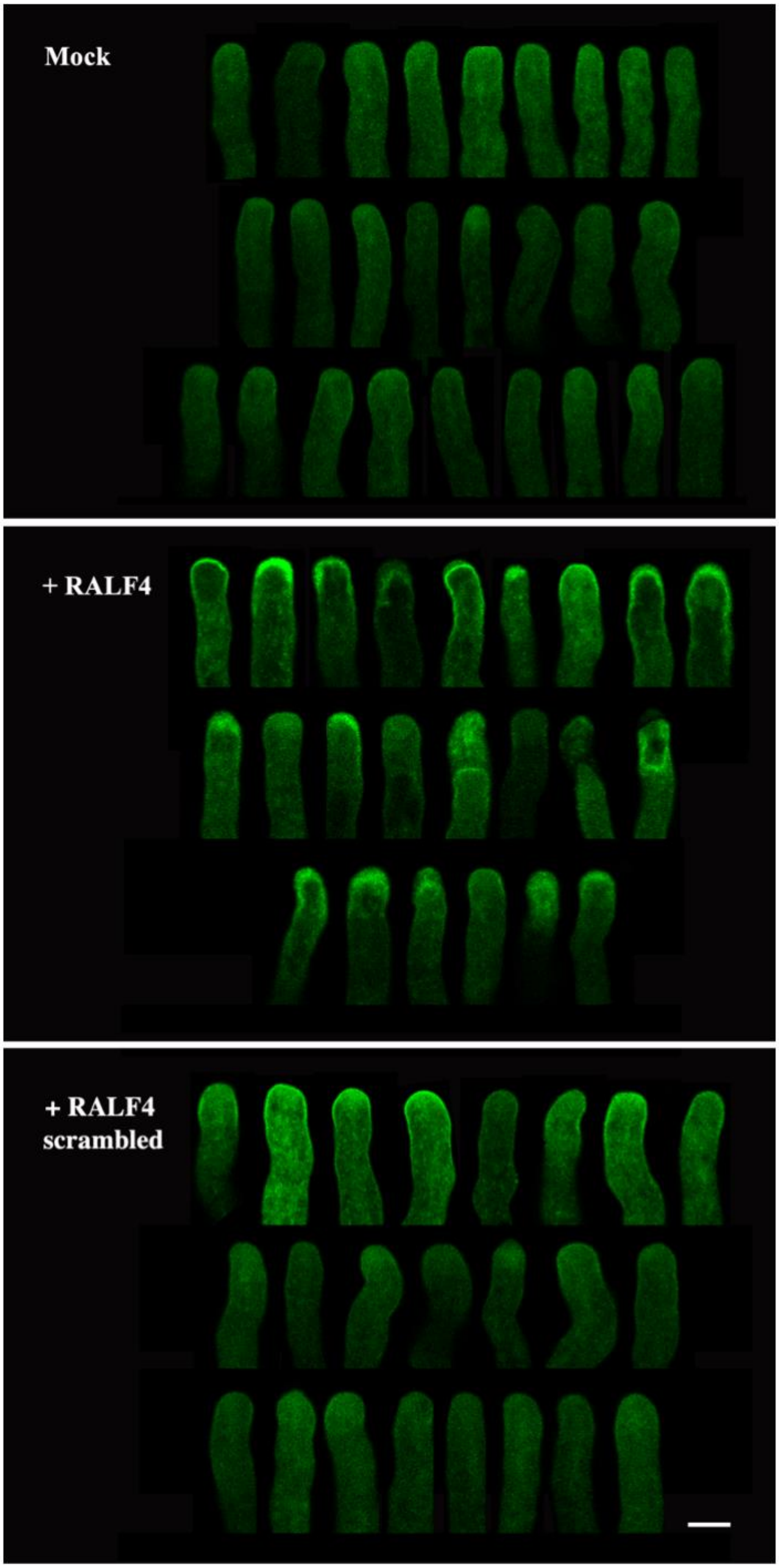
Mock
+ RALF4
+ RALF4
scrambled

Supplementary Figure S4: Localization of ANX1-YFP in pollen tubes. Multiple pollen tubes of *anx1 anx2* expressing *ANX1-YFP* after the treatment with PGM (Mock), RALF4 or RALF4 scrambled. Scale bar: 10 µm.

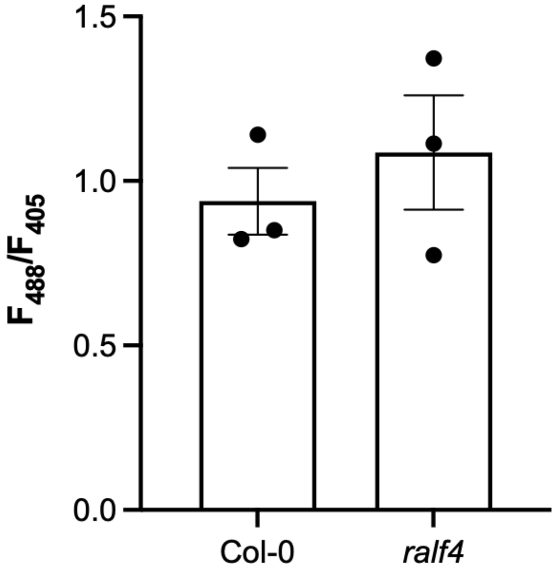

Supplementary Figure S5: Hydrogen peroxide levels in Col-0 and *ralf4* mutant pollen tubes. Data is shown as mean ± SEM of F_{488}/F_{405} ratio for three independent experiments with 13 pollen tubes each. Fluorescence in a 4 µm circle ROI in the tube tips was measured every 3 seconds for 3 minutes, for 6 to 12 (Col-0) and 6 to 9 (*ralf4*) pollen tubes per experiment. There is no significant difference between the genotypes according to the *t Student test.*

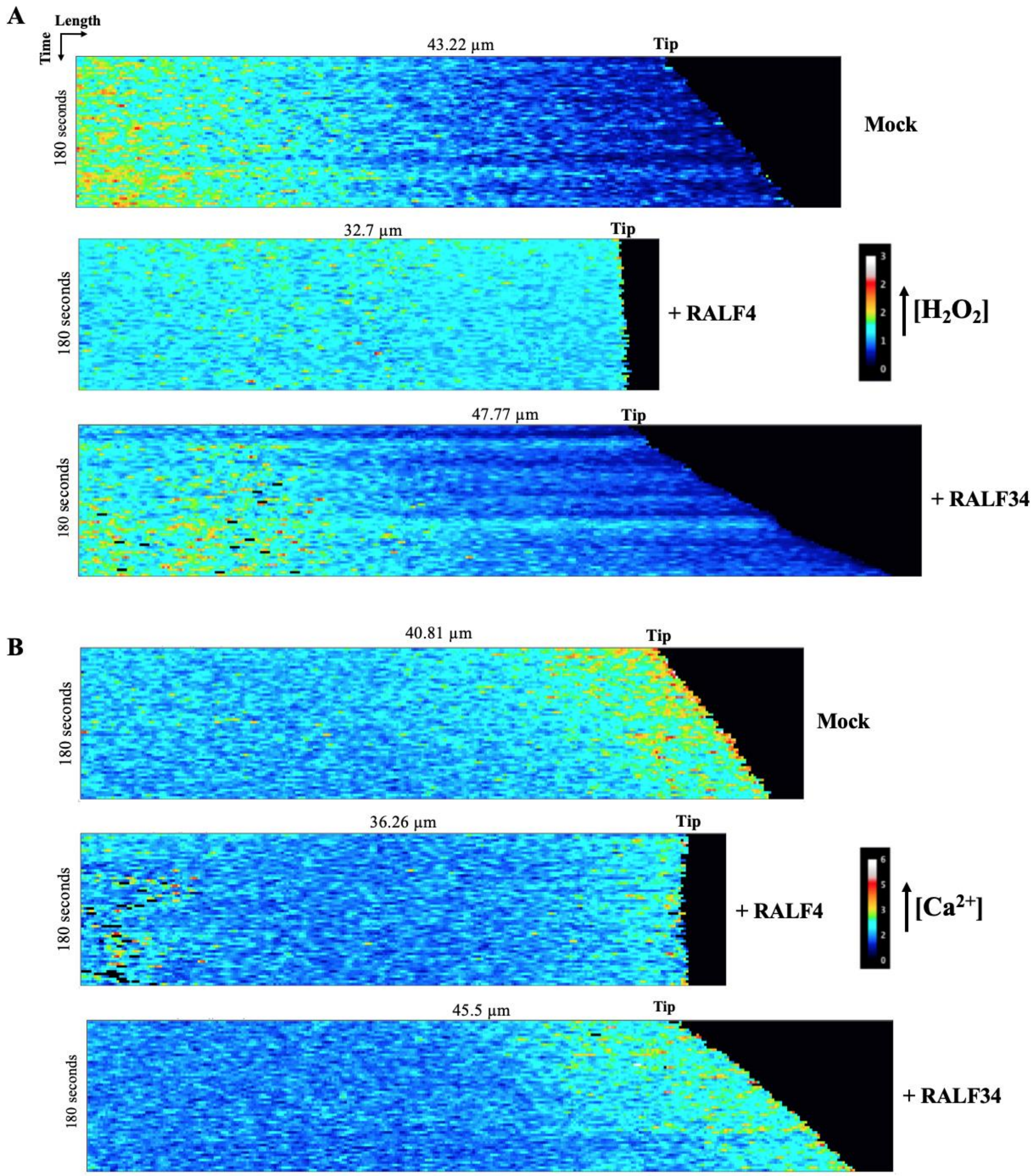

Supplementary Figure S6: H_2O_2 and Ca^{2+} levels over time along the tube after RALF treatments. Kymographs of representative pollen tubes expressing HyPer (**A**) and YC3.6 (**B**) treated with PGM (Mock) and 250 nM RALF4 and RALF34. Fluorescence was measured along a longitudinal line from the basal region to the tip (the length of the line is indicated above each kymograph), following its course along time (180 seconds).

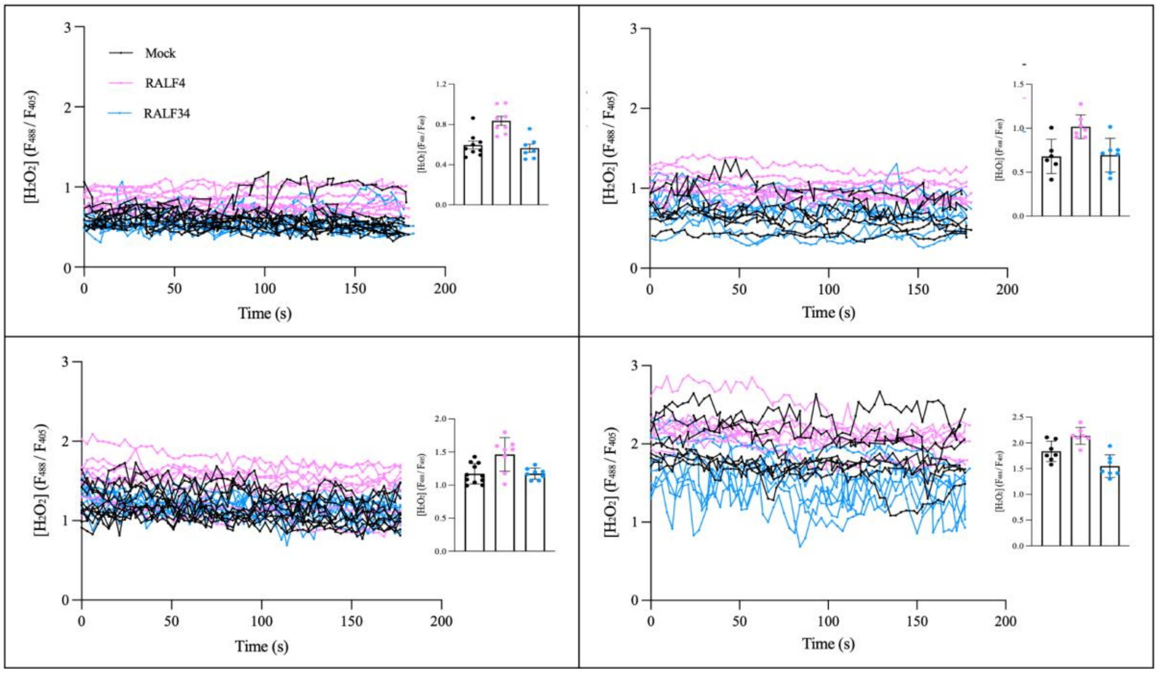

Supplementary Figure S7: Raw data for Figure 4, which details the cytoplasmic hydrogen peroxide levels in all mock and RALF4/34-treated pollen tubes monitored over four independent experiments. Cytoplasmic H_2O_2 levels at the tip of pollen tubes treated with PGM ("mock") (black), RALF4 (pink), and RALF34 (blue) over time. The mean ± SD for each pollen tube across each independent experiment is also shown.

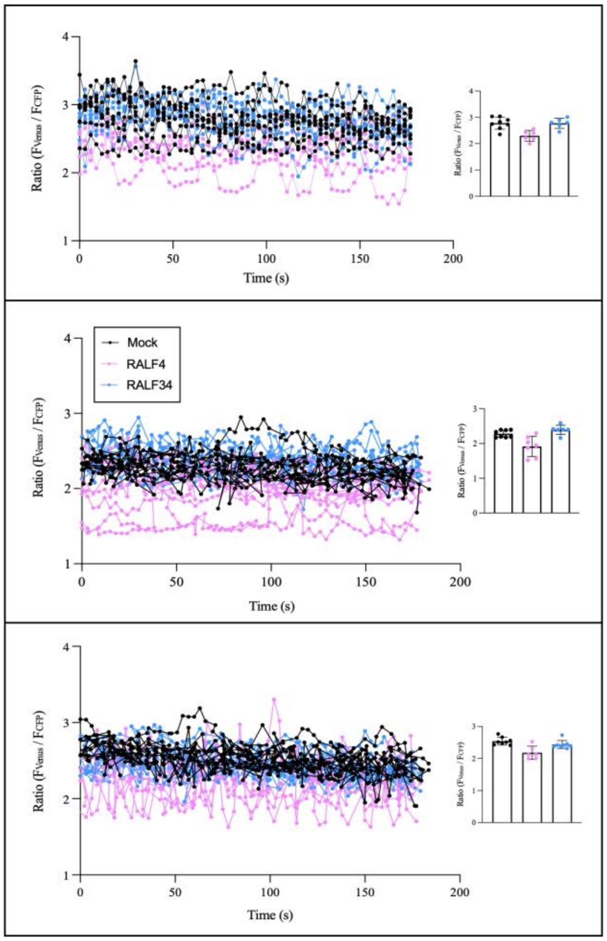

Supplementary Figure S8: Raw data for Figure 5, which details the cytoplasmic calcium levels in all mock and RALF4/34-treated pollen tubes monitored over three independent experiments. Cytoplasmic Ca^{2+} levels at the tip of pollen tubes treated with PGM ("mock") (black), RALF4 (pink), and RALF34 (blue) over time. The mean ± SD for each pollen tube across each independent experiment is also shown.

Supplementary Table S1

**Name**	**Sequence**
**RALF4/TAMRA-RALF4**	ARGRRYIGYDALKKNNVPCSRRGRSYYDCKKRRRNNPYRRGCSAITHCYRYAR
**RALF4 scrambled**	PTYNSCRRKCKRDRGYAARRYKRYRYVNADIKRNHSGYPCRICSRLYGRN
**RALF34**	YWRRTKYYISYGALSANRVPCPPRSGRSYYTHNCFRARGPVHPYSRGCSSITRCR R

Amino acid sequence of the synthetic peptides used in this work: RALF4, TAMRA-RALF4 (conjugated to 5-TAMRA, N-terminal), RALF4 scrambled and RALF34.

Video S1: Wild-type growing pollen tube after the addition of TAMRA-RALF4. The tube was recorded for approximately 7 minutes (1 snap per second) and is shown with a higher playback speed.

Video S2: **Wild-type growing pollen tubes before (left panel) and after (right panel) the addition of RALF4 peptide.** The tubes were recorded for 216 seconds in total (snap every 12 seconds) in both videos, which are shown with a higher playback speed.

Videos S3 A and B: **Wild-type growing pollen tube expressing cytosolic HyPer after the addition of PGM (Mock) (A) and RALF4 (B).** Bright field (left panels) and F_{488}/F_{405} ratio (right panels) are shown. The tubes were recorded for 3 minutes and shown with a higher playback speed.

Videos S4 A and B: **Wild-type growing pollen tube expressing YC3.6 after the addition of PGM (Mock) (A) and RALF4 (B)**. Bright field (left panels) and F_{488}/F_{405} ratio (right panels) are shown. The tubes were recorded for 3 minutes and shown with a higher playback speed.
