# Supplementary figures and images for: Arabidopsis RALF4 Rapidly Halts Pollen Tube Growth by Increasing ROS and Decreasing Calcium Cytoplasmic Tip Levels
Source: Biomolecules. 2024 Oct 29;14(11):1375. doi: 10.3390/biom14111375 (PMC11591785; doi:10.3390/biom14111375)

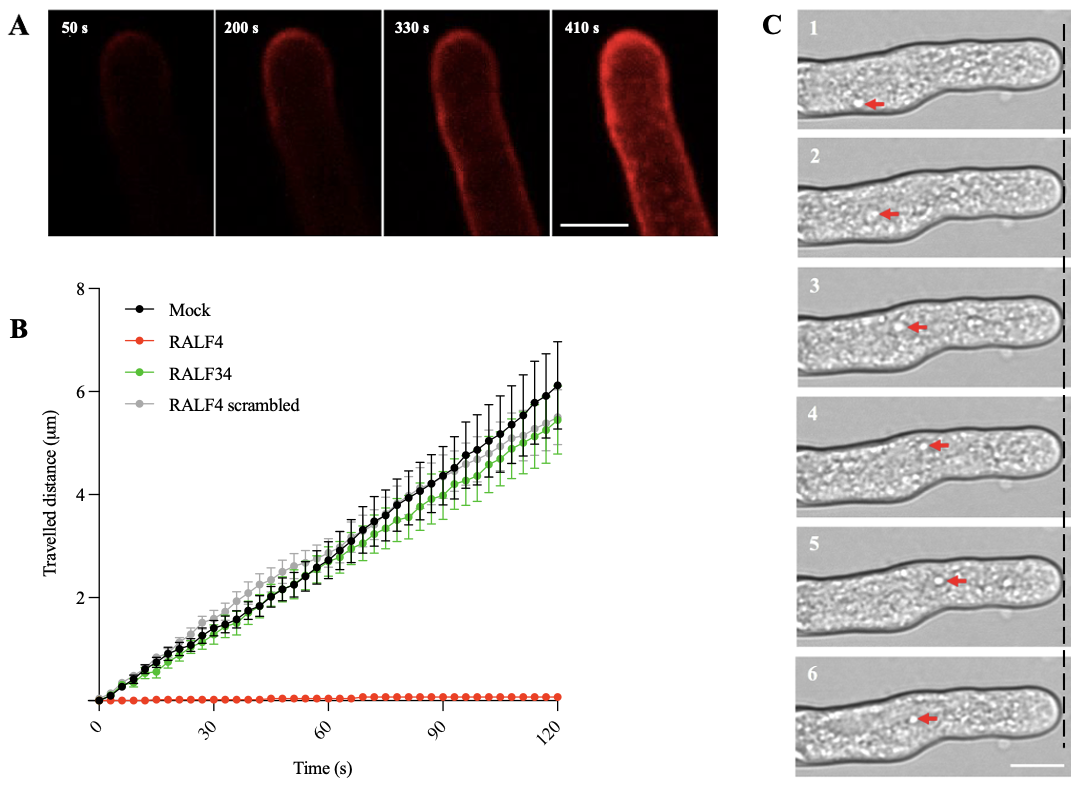

Supplement: Supplementary file 1 [file biomolecules-14-01375-s001.zip › figure S2.tiff]

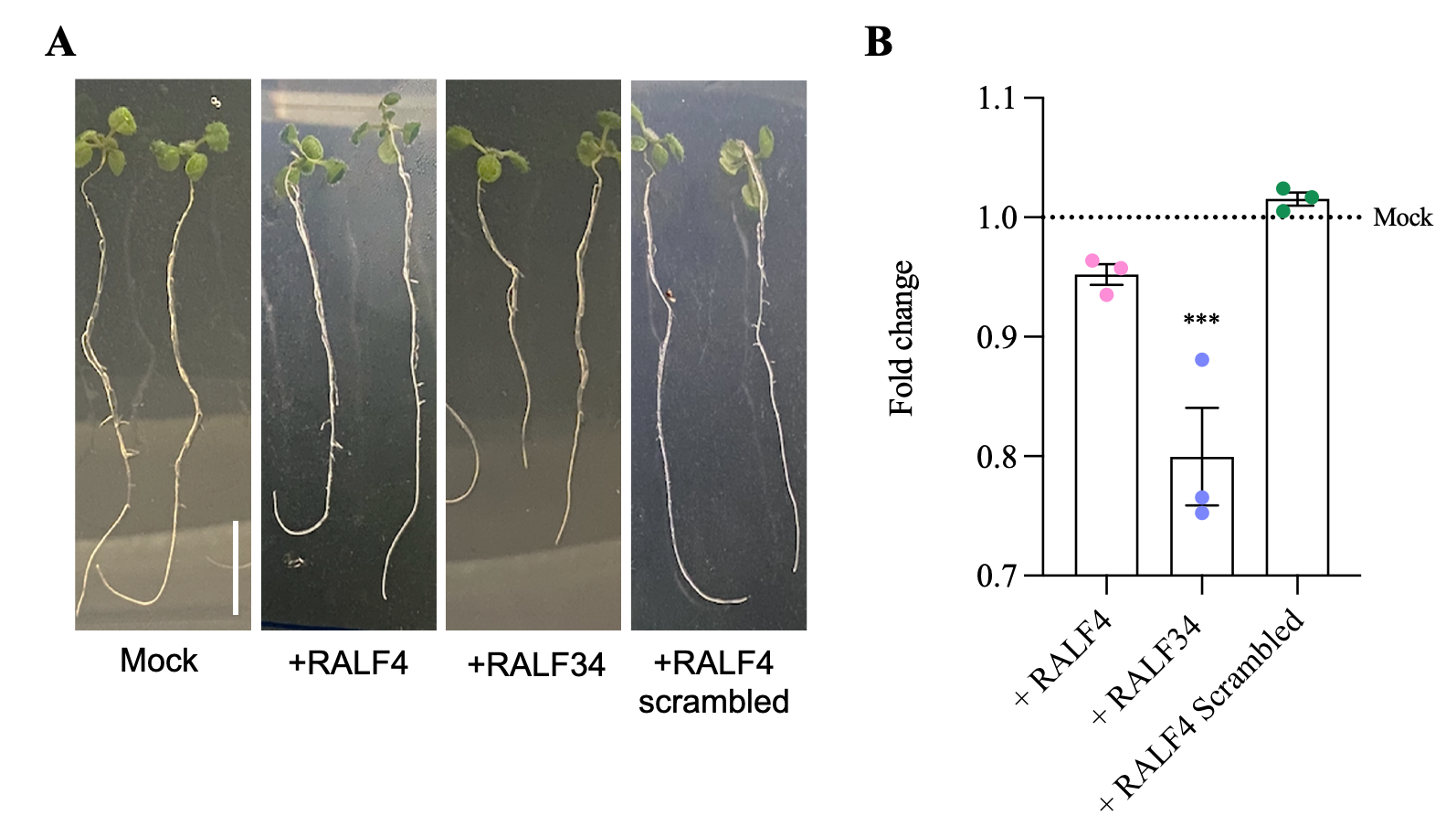

Supplement: Supplementary file 1 [file biomolecules-14-01375-s001.zip › figure s3.tiff]

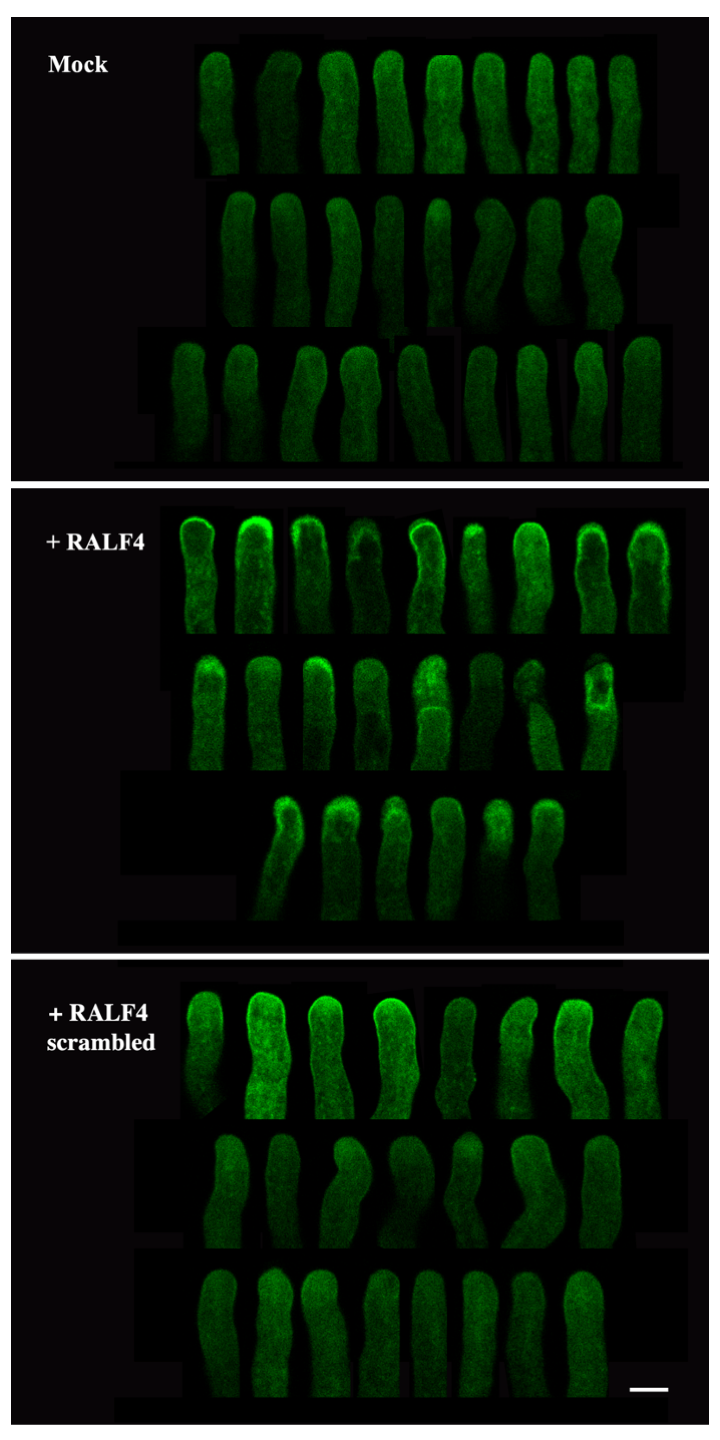

Supplement: Supplementary file 1 [file biomolecules-14-01375-s001.zip › figure S4.tiff]

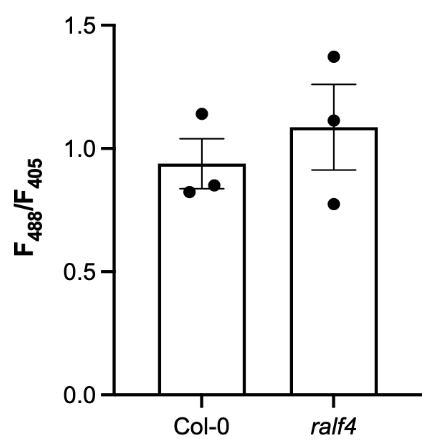

Supplement: Supplementary file 1 [file biomolecules-14-01375-s001.zip › figure s5.tiff]

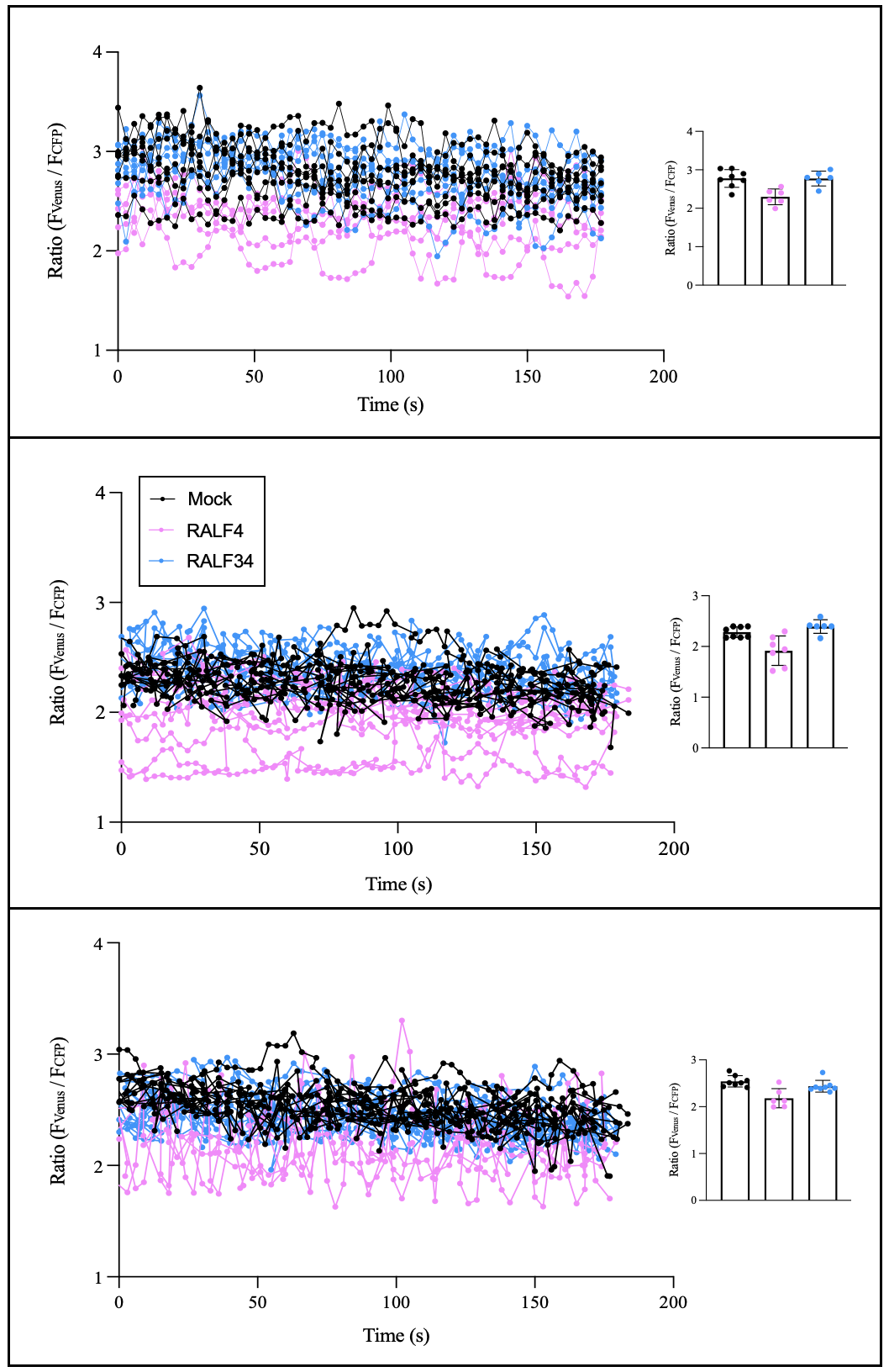

Supplement: Supplementary file 1 [file biomolecules-14-01375-s001.zip › figure S8.tiff]

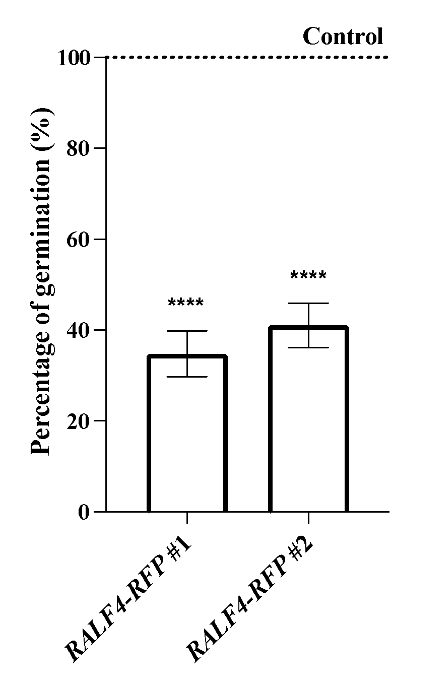

Supplement: Supplementary file 1 [file biomolecules-14-01375-s001.zip › figureS1.tiff]

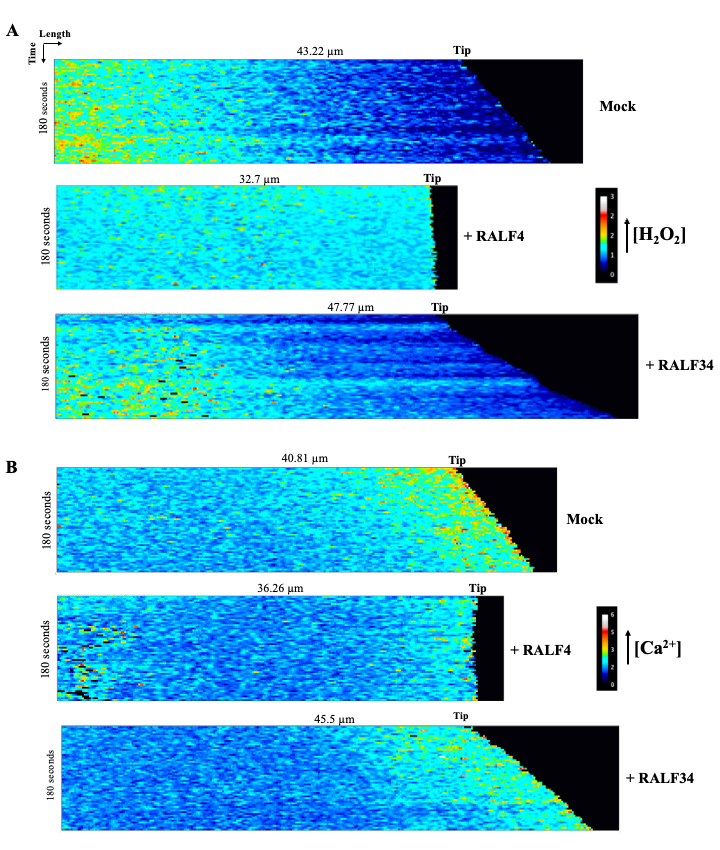

Supplement: Supplementary file 1 [file biomolecules-14-01375-s001.zip › figureS6.tiff]

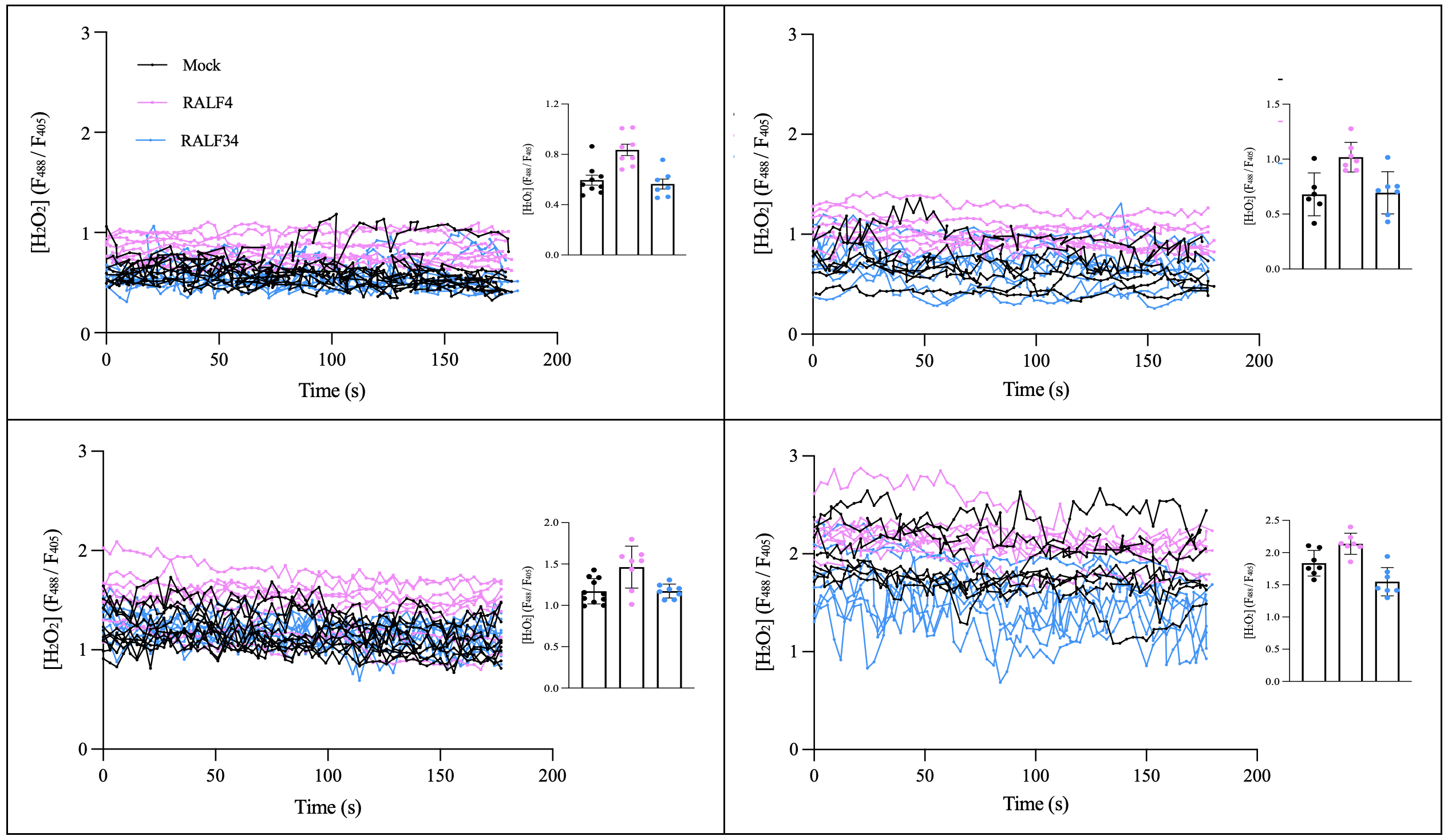

Supplement: Supplementary file 1 [file biomolecules-14-01375-s001.zip › figure_S7.tiff]
